# Supplementary material for: Serological responses to killed oral cholera vaccine (OCV) when given 4 years after initial receipt of OCV in Cameroon: A randomized controlled trial
Source: PLOS Glob Public Health. 2026 Apr 21;6(4):e0004913. doi: 10.1371/journal.pgph.0004913 (PMC13098963; doi:10.1371/journal.pgph.0004913)
Supplement: S1 Appendix — (DOCX) [file pgph.0004913.s003.docx]

Table A: **Comparison of Vibriocidal GMT and Seroconversion rates 4-6 days after a single dose of OCV depending on receipt of OCV four years earlier.**

| **Blood characteristics (outcomes) 4-6 days after the single dose administration** | **Cohort A Number of OCV doses exposed four year earlier** | | | **Cohort A Number of OCV doses exposed four year earlier** | |
| --- | --- | --- | --- | --- | --- |
|  | **Zero dose** | **Two doses** | **P.value (adjusted)** | **One or two doses** | **P.value (adjusted)** |
| **All age group** | **N=59** | **N=49** |  | **N=92** |  |
| **VGMT Inaba, [CI]+** | 15.1 [10;22.8] | 16.4 [10.4;25.8] | 0.7 | 13.9 [10.2;19.1] | 0.8 |
| **VGMT Ogawa, [CI]+** | 29.1 [18.5;45.8] | 51.6 [29.9;89] | 0.1 | 41.2 [28.6;59.3] | 0.2 |
| **VSCR Inaba, %[CI]*** | 23.7 [12.5;34.9] | 22.4 [10.3;34.6] | 0.9 (0.6) | 12 [5.2;18.7] | 0.1 (0.1) |
| **VSCR Ogawa, %[CI]*** | 15.3 [5.80;24.7] | 18.4 [7.10;29.6] | 0.7 (0.5) | 10.9 [4.4;17.4] | 0.4 (0.6) |
| **1-4 years** | **N=17** | **N=9** |  | **N=13** |  |
| **VGMT Inaba, [CI]+** | 17.7 [7.2;43.2] | 20 [5.10;77.8] | 0.6 | 13.8 [5.30;35.5] | 1 |
| **VGMT Ogawa, [CI]+** | 14.4 [7.10;29.5] | 31.7 [9.1;110.8] | 0.2 | 24.8 [8.7;70.4] | 0.4 |
| **VSCR Inaba, % [CI]*** | 29.4 [5.30;53.6] | 33.3 [0;71.8] | 0.8(0.9) | 23.1 [5.30;49.6] | 0.7 (0.7 ) |
| **VSCR Ogawa, % [CI]*** | 29.4 [5.30;53.6] | 44.4 [3.9;85] | 0.4(0.3) | 30.8 [5.3;59.8] | 0.9 (0.6 ) |
| **5-14 years** | **N=22** | **N=22** |  | **N=42** |  |
| **VGMT Inaba, [CI]+** | 14.1 [7;28.5] | 10 [5.7;17.7] | 0.3 | 9.8 [6.7;14.3] | 0.3 |
| **VGMT Ogawa, [CI]+** | 24.9 [14.4;43.1] | 30.1 [14.4;63.1] | 0.9 | 33.4 [21.1;52.9] | 0.6 |
| **VSCR Inaba, % [CI]*** | 22.7 [3.7;41.7] | 18.2 [0.7;35.7] | 0.7(0.3) | 9.5 [3.7;18.8] | 0.1 (0 ) |
| **VSCR Ogawa, % [CI]*** | 18.2 [0.7;35.7] | 13.6 [0;29.2] | 0.7(0.2) | 7.1 [0.7;15.3] | 0.2 (0 ) |
| **> 14 years** | **N=20** | **N=18** |  | **N=37** |  |
| **VGMT Inaba, [CI]+** | 14.1 [6.80;29.2] | 27.2 [11.6;64] | 0.2 | 20.8 [11.6;37.1] | 0.4 |
| **VGMT Ogawa, [CI]+** | 62.8 [22.5;175.3] | 127 [46.2;348.7] | 0.3 | 62.7 [32.2;122.3] | 0.9 |
| **VSCR Inaba, % [CI]*** | 20 [.8;39.2] | 22.2 [0.9;43.5] | 0.9(0.2) | 10.8 [0.8;21.3] | 0.3 (1 ) |
| **VSCR Ogawa, % [CI]*** | 0 [0;0] | 11.1 [0;27.2] | 0.1 | 8.1 [0;17.3] | 0.2 |

+ P.value are from Wilcoxon test, * P.value are from chi-square, VGMT= Vibriocidal Geometric mean titer, VSCR= vibriocidal seroconversion rate. The logistic regression model has been used for the adjustment with seroconversion status as dependent variable, vaccination status in 2017 as main independent variable and other variables (Sex, Age group, floor material, using improved toilet facility, weight, and height) as adjustment variables (age group was considered as adjustment variable for all age).

Table B: **Comparison of Vibriocidal GMT and Seroconversion rates 9-11 days after a single dose of OCV depending on receipt of OCV four years earlier.**

| **Blood characteristics (outcomes) 9-11 days after the single dose administration** | **Cohort A Number of OCV doses exposed four year earlier** | | | **Cohort A Number of OCV doses exposed four year earlier** | |
| --- | --- | --- | --- | --- | --- |
|  | **Zero dose** | **Two doses** | **P.value (adjusted)** | **One or two doses** | P.value (adjusted) |
| **All age group** | N=59 | N=47 |  | N=87 |  |
| **VGMT Inaba, [CI]+** | 63.2 [40.7;98.3] | 88.7 [48.5;162.1] | 0.5 | 75.7 [49.6;115.3] | 0.7 |
| **VGMT Ogawa, [CI]+** | 87.9 [57.6;134.1] | 382 [233.5;624.8] | 0 | 246 [172.1;351.6] | 0 |
| **VSCR Inaba, %[CI]*** | 62.7 [50;75.4] | 68.1 [54.3;81.9] | 0.6 (0.4) | 64.4 [54.1;74.6] | 0.8 (0.6) |
| **VSCR Ogawa, %[CI]*** | 61 [48.2;73.8] | 70.2 [56.6;83.8] | 0.3 (0.2) | 65.5 [55.3;75.7] | 0.6 (0.3) |
| **1-4 years** | N=19 | N=8 |  | N=11 |  |
| **VGMT Inaba, [CI]+** | 96 [38.6;238.6] | 320 [64;1600.1] | 0.2 | 150.2 [35.8;630.6] | 0.6 |
| **VGMT Ogawa, [CI]+** | 62 [26.1;147.1] | 380.5 [103.2;1403.3] | 0 | 248.7 [71;871.7] | 0.1 |
| **VSCR Inaba, % [CI]*** | 73.7 [51.9;95.5] | 100 [100;100] | 0.1 | 90.9 [51.9;111.2] | 0.3 (1 ) |
| **VSCR Ogawa, % [CI]*** | 73.7 [51.9;95.5] | 100 [100;100] | 0.1 | 90.9 [51.9;111.2] | 0.3 (0.6 ) |
| **5-14 years** | N=20 | N=21 |  | N=42 |  |
| **VGMT Inaba, [CI]+** | 65 [32.6;129.7] | 80 [32.2;198.5] | 0.8 | 85.5 [47.4;154.1] | 0.6 |
| **VGMT Ogawa, [CI]+** | 121.3 [74.5;197.3] | 330.7 [144.8;755.2] | 0 | 245.7 [149.4;404.3] | 0 |
| **VSCR Inaba, % [CI]*** | 60 [36.5;83.5] | 71.4 [50.4;92.5] | 0.4(1) | 73.8 [36.5;87.7] | 0.3 (0.7 ) |
| **VSCR Ogawa, % [CI]*** | 80 [60.8;99.2] | 71.4 [50.4;92.5] | 0.5(0.5) | 66.7 [60.8;81.5] | 0.3 (0.3 ) |
| **> 14 years** | N=20 | N=18 |  | N=34 |  |
| **VGMT Inaba, [CI]+** | 41.4 [18.9;90.7] | 56.6 [20.4;156.7] | 0.7 | 52.1 [25.8;105.2] | 0.7 |
| **VGMT Ogawa, [CI]+** | 88.8 [36.3;217.2] | 452.5 [200.8;1020] | 0 | 245.5 [133;453.2] | 0.1 |
| **VSCR Inaba, % [CI]*** | 55 [31.1;78.9] | 50 [24.4;75.6] | 0.8(0.7) | 44.1 [31.1;61.7] | 0.4 (0.5 ) |
| **VSCR Ogawa, % [CI]*** | 30 [8;52] | 55.6 [30.1;81] | 0.1(0.3) | 55.9 [8;73.5] | 0.1 (0.1 ) |

+ P.value from Wilcoxon test, * P.value from chi-square, VGMT= Vibriocidal Geometric mean titer, VSCR= vibriocidal seroconversion rate The logistic regression model has been used for the adjustment with seroconversion status as dependent variable , vaccination status in 2017 as main independent variable and other variables (Sex, Age group, floor material, using improved toilet facility ,weight, and height) as adjustment variables (age group has been included only at the global level).

Table C: **Comparison of Vibriocidal GMT and Seroconversion rates 28-30 days after the first dose of OCV arm A and arm B.**

| **Participants** | **Cohort A and cohort B immune response 28-30 days after the first dose administration** | | |
| --- | --- | --- | --- |
|  | **Arm A** | **Arm B** | **P.value (adjusted)** |
| **All age group** | **N=147** | **N=150** |  |
| **All age group** | 33.6 [25.6;44.1] | 43.9 [32.6;59.2] | 0.2 |
| **VGMT Inaba, [CI]+** | 71.4 [54;94.5] | 79.3 [59.9;104.9] | 0.6 |
| **VGMT Ogawa, [CI]+** | 49.7 [41.5,57.8] | 50.3 [42.2,58.5] | 0.9 (0.8) |
| **VSCR Inaba, %[CI]*** | 33.3 [25.6,41] | 43.3 [35.3,51.4] | 0.1 (0.1) |
| **VSCR Ogawa, %[CI]*** | **N=31** | **N=33** |  |
| **1-4 years** | 26.7 [14.6;49] | 20 [11.3;35.3] | 0.5 |
| **VGMT Inaba, [CI]+** | 26.7 [15.7;45.5] | 25.2 [15.4;41.1] | 1 |
| **VGMT Ogawa, [CI]+** | 45.2 [26.6,63.7] | 31.3 [14.3,48.2] | 0.3 (0.2) |
| **VSCR Inaba, % [CI]*** | 38.7 [20.5,56.9] | 45.5 [27.5,63.4] | 0.6 (0.6) |
| **VSCR Ogawa, % [CI]*** | **N=61** | **N=63** |  |
| **5-14 years** | 33 [21.4;50.8] | 73.3 [45.6;117.7] | 0 |
| **VGMT Inaba, [CI]+** | 87.6 [58.6;130.9] | 138.7 [93;206.7] | 0.1 |
| **VGMT Ogawa, [CI]+** | 50.8 [37.9,63.7] | 63.5 [51.3,75.7] | 0.2 (0.1) |
| **VSCR Inaba, % [CI]*** | 41 [28.3,53.7] | 50.8 [38.1,63.5] | 0.3 (0.2) |
| **VSCR Ogawa, % [CI]*** | N=55 | N=54 |  |
| **> 14 years** | 39 [24.7;61.5] | 38.5 [23.6;62.8] | 1 |
| **VGMT Inaba, [CI]+** | 99.1 [60.3;162.9] | 83.1 [51;135.5] | 0.7 |
| **VGMT Ogawa, [CI]+** | 50.9 [37.3,64.5] | 46.3 [32.6,60] | 0.6 (0.6) |
| **VSCR Inaba, % [CI]*** | 21.8 [10.6,33.1] | 33.3 [20.3,46.3] | 0.2 (0.1) |

+ P.value are from Wilcoxon test, * P.value are from chi-square, VGMT= Vibriocidal Geometric mean titer, VSCR= vibriocidal seroconversion rate. The logistic regression model has been used for the adjustment with seroconversion status as dependent variable, vaccination status in 2017 as main independent variable and other variables (Sex, Age group, floor material, using improved toilet facility, weight, and height) as adjustment variables (age group was considered as adjustment variable for all age).
