# Supplementary material for: Serological responses to killed oral cholera vaccine (OCV) when given 4 years after initial receipt of OCV in Cameroon: A randomized controlled trial
Source: PLOS Glob Public Health. 2026 Apr 21;6(4):e0004913. doi: 10.1371/journal.pgph.0004913 (PMC13098963; doi:10.1371/journal.pgph.0004913)
Supplement: Attachment — Submitted filename: PGPH-D-25-01336_R1_31Dec2025.pdf [file pgph.0004913.s007.pdf]

PLOS Global Public Health

Serological responses to killed oral cholera vaccine (OCV) when given 4 years after initial receipt of OCV in Cameroon: A randomized controlled trial

--Manuscript Draft--

**Manuscript Number:**	PGPH-D-25-01336R1
**Article Type:**	Clinical Trial
**Full Title:**	Serological responses to killed oral cholera vaccine (OCV) when given 4 years after initial receipt of OCV in Cameroon: A randomized controlled trial
**Short Title:**	Oral Cholera Vaccine booster dose serological response in Cameroon
**Corresponding Author:**	Jerome Ateudjieu, Ph.D<br>M.A. SANTE<br>Yoaunde, Center CAMEROON
**Order of Authors:**	Jerome Ateudjieu, Ph.D
	Ketina Hirma Tchio-Nighie, MPH
	Etienne Guenou
	Carrel Fokou
	Collins Buh Nkum
	Landry Beyala Bita'a
	Winny Dora Ateudjieu-Kenfack
	Sabine Nanfak
	Amanda K Debes
	David A Sack
**Keywords:**	cholera; Cholera vaccine; booster dose; Cameroon
**Abstract:**	Background: Oral cholera vaccine (OCV) reduces the rates of cholera in populations at risk. Two doses are recommended for people living in hotspot areas as a preventative intervention. Since the vaccine is known to provide protection for 3 to 5 years, revaccination for these areas is warranted to continue to provide immunity. When revaccination is considered, there is a need for evidence guiding whether revaccination should use two doses or whether a single dose may be sufficient because this may induce a booster response. An OCV campaign was conducted in Mogode, Cameroon in 2017 which provided an opportunity to determine if this earlier OCV campaign would induce a booster response if revaccination was conducted four years later.<br>Methods. 350 people living in Mogode Cameroon, who received 0, 1 or 2 doses of OCV four years earlier, were stratified into age groups, <5, 5-14, and >14 and were randomized into two arms. Arm A received a single dose and Arm B received two doses with the second dose given 14 days after the first. We aimed to determine if people who had received OCV four years earlier responded with a booster immune response (as suggested by a rapid rise in vibriocidal titer (by day 4 to 6)) compared to those who had not received OCV earlier. Serum, was collected at baseline for both groups. For Arm A, additional serum samples were obtained at day 4, 9, 14 and 28 following the first dose. For Arm B, additional serum samples were obtained on these same days, but after the second dose. Vibriocidal titers were determined for each of the serum samples.<br>Results. Nine days after the OCV, vibriocidal GMT significantly increased in each of study arms with a response rate of 61 to 70%; however, an early vibriocidal response on day 4-6 was rare (22%) and were the same in those who had received vaccine four years earlier and those who did not. Ogawa, but not Inaba, vibriocidal responses were higher on day 9 for those who had been vaccinated earlier, but vibriocidal titers during subsequent timepoints were similar in all study arms. Four to six days following the second dose (Arm B), there was no evidence of an early higher booster response compared to those of Arm A.<br>Conclusion. An increase in the proportion of subjects showing an early vibriocidal

Powered by Editorial Manager® and ProduXion Manager® from Aries Systems Corporation

	serological response was not seen among people who received OCV 4 years earlier compared to those who did not receive. The tendency was observed in each age group. Pour le serotype Ogawa, le peak vibriocidal serological response was higher in people vaccinated four year earlier compared to those not previously vaccinated. The tendence was observed in Arm A and Arm B. Further studies are needed to confirm these findings and guide the booster vaccination protocol using the OCV vaccine.
Additional Information:	
Question	Response
**Financial Disclosure**<br><br>Enter a financial disclosure statement that describes the sources of funding for the work included in this submission and the role the funder(s) played. This includes grants and any commercial funding of the work or authors.<br><br>This statement will be typeset if the manuscript is accepted for publication.<br><br>*Please review the submission guidelines and the instructions link below for detailed requirements and guidance.*	This work was supported by a grant from the Wellcome Trust to JA (grant number: 215663/Z/19/Z) and a grant from the National Institute of Allergy and Infectious Disease to DAS (5R01AI123422). The Wellcome Trust provided suggestions as to the study design, but neither funder had any role in data collection and analysis, decision to publish, or preparation of the manuscript.
**Competing Interests**<br><br>On behalf of all authors, disclose any competing interests that could be perceived to bias this work.<br><br>This statement will be typeset if the manuscript is accepted for publication.<br><br>*Please review the instructions link below and PLOS Global Public Health's competing interests policy to determine what information must be disclosed at submission.*	The authors have declared that no competing interests exist.
**Data Availability**<br><br>Before publication, Authors are required to make fully available and without restriction all data underlying their	Data presented or used for the analysis in the present manuscript are available as "Supporting Information File 1"

findings. Please see our PLOS Data Policy page for detailed information on this policy.<br><br>A **Data Availability Statement**, detailing where the data can be accessed, is required at first submission. Insert your Data Availability Statement in the box below.<br><br>Please see the data reporting section of our submission guidelines for instructions on what you need to include in your Data Availability Statement.<br><br>This statement will be typeset if the manuscript is accepted for publication.<br><br>*PLOS allows rare exemptions to address legal and ethical concerns. If you have legal or ethical restrictions, please use the box below to detail these in full sentences for the Journal team to consider.*	

Powered by Editorial Manager® and ProduXion Manager® from Aries Systems Corporation

Manuscript

Click here to access/download;Manuscript;Manuscript Nov24.docx

Title: Serological responses to killed oral cholera vaccine (OCV) when given 4 years after initial receipt of OCV in Cameroon: A randomized controlled trial.

Short title: Oral Cholera Vaccine booster dose serological response in Cameroon

Jerome Ateudjieu[1,2,3*], Ketina Hirma Tchio-Nighie[1,2], Etienne Guenou[1,2,6], Carrel Fokou[1], Collins Buh Nkum[1], Landry Beyala Bita'a[1], Winny Dora Ateudjieu-Kenfack[4,5], Sabine Nanfak[1], Amanda K Debes[7], David A Sack[7]

Affiliations

[1]M.A. SANTE, Yaounde, Cameroon

[2] Faculty of Medicine and Pharmaceutical Sciences, University of Dschang, Dschang, Cameroon.

[3]Clinical Research Unit, Division of Health Operations Research, Ministry of Public Health, Cameroon. Yaounde, Cameroon

[4] Department Microbiology, Faculty of Sciences University of Yaounde 1, Yaounde, Cameroon

[5]*Center of Research on Emerging and Reemerging Diseases, Yaounde, Cameroon*.

[6]National Public Health Laboratory, Ministry of Public Health, Yaounde, Cameroon.

[7]Johns Hopkins Bloomberg School of Public Health, Baltimore, Maryland. USA.

*Corresponding author

Jerome Ateudjieu: jateudj@masante-cam.org

Abstract

Background. Oral cholera vaccine (OCV) reduces the rates of cholera in populations at risk. Two doses are recommended for people living in hotspot areas as a preventative intervention. Since the vaccine is known to provide protection for 3 to 5 years, revaccination for these areas is warranted to continue to provide immunity. When revaccination is considered, there is a need for evidence guiding whether revaccination should use two doses or whether a single dose may be sufficient because this may induce a booster response. An OCV campaign was conducted in Mogode, Cameroon in 2017 which provided an opportunity to determine if this earlier OCV campaign would induce a booster response if revaccination was conducted four years later.

Methods. 350 people living in Mogode Cameroon, who received 0, 1 or 2 doses of OCV four years earlier, were stratified into age groups, <5, 5-14, and >14 and were randomized into two arms. Arm A received a single dose and Arm B received two doses with the second dose given 14 days after the first. We aimed to determine if people who had received OCV four years earlier responded with a booster immune response (as suggested by a rapid rise in vibriocidal titer (by day 4 to 6)) compared to those who had not received OCV earlier. Serum, was collected at baseline for both groups. For Arm A, additional serum samples were obtained at day 4, 9, 14 and 28 following the first dose. For Arm B, additional serum samples were obtained on these same days, but after the second dose. Vibriocidal titers were determined for each of the serum samples.

Results. Nine days after the OCV, vibriocidal GMT significantly increased in each of study arms with a response rate of 61 to 70%; however, an early vibriocidal response on day 4-6 was rare (22%) and were the same in those who had received vaccine four years earlier and those who did not. Ogawa, but not Inaba, vibriocidal responses were higher on day 9 for those who had been vaccinated earlier, but vibriocidal titers during subsequent timepoints were similar in all study arms. Four to six days following the second dose (Arm B), there was no evidence of an early higher booster response compared to those of Arm A.

Conclusion. An increase in the proportion of subjects showing an early vibriocidal serological response was not seen among people who received OCV 4 years earlier compared to those who did not receive. The tendency was observed in each age group. Pour le serotype Ogawa, le peak vibriocidal serological response was higher in people vaccinated four year earlier compared to those not previously vaccinated. The tendence was observed in Arm A and Arm B. Further studies are needed to confirm these findings and guide the booster vaccination protocol using the OCV vaccine.

Trial registration: Pan African Clinical Trials Registry under the reference PACTR202102660004195.

Background

Cholera is currently one of the most alarming diseases with epidemic potential because of frequent outbreaks in vulnerable populations, rapid spread, high case-fatality rate, and negative impact on the performance of health systems. (1–3). Oral Cholera Vaccine (OCV) vaccination is one of the most effective responses to prevent cholera transmission, propagation, morbidity and mortality (4–6). While both killed whole cell and live attenuated vaccines have been licensed, only the killed oral whole cell vaccine is used through the global stockpile (7,8). This vaccine is recommended by the World Health Organization (WHO) in hotspots of cholera endemic countries, during humanitarian crises with high a risk of cholera, and during cholera outbreaks (9–11). Since the duration of protection of OCV is estimated to be 3 to 5 years, the WHO recommends that revaccination may be needed after 3 years where there is continued risk of cholera (12). To date, the vaccine manufacturers have not issued any recommendations for booster vaccination for the killed whole cell cholera vaccine.

Two studies examined the response following a repeated (booster) dose. A study in Bangladesh found that children age <5 years had a higher serological response to a single dose of OCV as measured by geometric mean vibriocidal titers if they had received the vaccine three years earlier, but other age groups (age >5 years and adults) did not show this enhanced response (13). By contrast, a study from India did not find a difference in response to OCV when two doses of vaccine was given 5 years after

the first vaccination (14). Neither of these studies examined the kinetics of the immune response, such as a rapid antibody response in the first week that may indicate a true booster response (15–17). Because of an outbreak in the area, the Cameroon Ministry of Public Health and its partners carried out an OCV vaccination campaign in 2017 in Mogode Health District located in the Far North region of Cameroon. This was a two rounds immunization campaign using Shanchol that targeted people aged ≥1 year old. A coverage survey conducted between the two rounds and after the second round estimated that 80.1% of the population had received two doses and 4.3% had received one dose (18). Since the campaign, there have been no reports of cholera between 2017 and 2021 nor has there been any administration of cholera vaccinations by the Ministry of Health that may have confounded this analysis. This previous exposure of the populations of Mogode Health district to OCV vaccination provided an opportunity to explore the immune response of a booster OCV vaccination to answer the following questions. 1) When given OCV, do people who had received two OCV doses four years earlier develop a rapid (within 4 to 6 days) serum vibriocidal response more commonly than those who did not receive OCV four years earlier. 2) When given OCV, is the geometric mean titer (GMT) vibriocidal in each age group (1-4, 5-14, ≥15) superior in people who received OCV four years earlier compared to those who did not receive OCV four years earlier? 3) When given OCV, is the serum vibriocidal responses in children inferior to the response in older age groups? 4) Do the immune responses after one or two doses OCV administration, differ in people exposed to one or two doses of OCV earlier?

It is anticipated that the findings from this study will help guide use of OCV when given to groups of people who were previously vaccinated. If persons previously vaccinated show evidence of a booster response, then it would seem that a single dose would provide sufficient immunity and that a second dose would not be needed. However, if a booster dose is not observed, this would suggest that such groups should be provided vaccine in the same way as groups that had not previously received vaccine.

Materials and Methods

Trial registration

The study protocol was registered in Pan African Clinical Trials Registry under the reference PACTR202102660004195(https://pactr.samrc.ac.za/TrialDisplay.aspx?TrialID=14625).

Study design

Individuals leaving in Mogode Health District aged four years and above and who had received zero, one or two doses of OCV during the 2017 OCV campaign were eligible to participate. Those fulfilling inclusion criteria were stratified by age (4 years, 5-14 years, ≥15 years) and 2017 OCV exposure status (zero dose, one dose, two doses) and randomly assigned to receive one dose of OCV at day zero (Arm A) or two doses of OCV at day 0 and 14 (Arm B). Serum samples were collected day 4-6, 9-12, 14-16 and 28-30 following administrations of dose 1 in arm A and on day 4-6, 9-12, 14-16 and 28-30 after the second dose (arm B) to determine and compare the overall serum vibriocidal responses and by age group according to 2017 pre-vaccination exposure status, and to compare the vibriocidal response per study arm.

Setting

The study was conducted in Mogode health district located in the Far North region of Cameroon. Mogode health district benefited from a two rounds OCV campaign in May and June 2017. The immunization coverage estimated from the end campaign survey was estimated for two, one and zero doses documented coverage at 80.1%, 4.3% and 3.8% respectively (18). Enrollment of participants for the present trial was conducted from February to June 2021. Between this campaign and the start of the study in 2021, no case of cholera was detected in Mogode health District or any of bordering health district by the surveillance system in place and no other campaign with OCV was conducted in the area during the interval. In the same period, no additional OCV vaccination activity was conducted in the Mogode Heath District or in any of bordering health District.

Participants

All individuals aged 1 year and above during the 2017 OCV vaccination campaign living in the Mogode health district and who received either two, one or zero doses of OCV during the 2017 campaign were eligible. We excluded all individuals who reported to have received OCV vaccines after the 2017 OCV campaign before the study period, women who were pregnant, persons enrolled in another study, those with a history of diarrhea during the seven days prior to first dose of vaccine (defined as ≥3 loose stools in 24 hours) or history of chronic diarrhea (lasting for more than 2 weeks in the past 6 months), those currently using laxatives, antacids, or other agents to lower stomach acidity, those with chromic health conditions, or immunosuppression and persons not willing to provide informed consent.

Sample size

To determine whether a rapid vibriocidal response following single dose OCV administration among those exposed to OCV four years earlier is superior to those exposed to zero doses four years earlier, we attempted to recruit 20 people for each age group (1-4, 5-14, ≥15 years old) in Arm A and the same number for Arm B. Among those who had received OCV four years earlier, we intended to evaluate each age group into those who had received zero, one dose or two doses earlier. Thus, total sample size was estimated to be 360 with 180 participants expected per study arm, including 60 per OCV vaccination status (0, 1 or 2 doses) distributed in 20 per age group. We hypothesized that an early vibriocidal response following single dose administration (Arm A) will occur among 25% of those in non-exposed group (zero OCV dose four years earlier) and will be increased to 75% in the exposed group (exposed to 2 OCV doses four years earlier) (confidence=95%, power=90%), that early immune response in arm A will not differ to that of Arm B and that the dropout rate in each study arm will be 10% or less.

Study procedures

Community involvement. With the help of local health authorities, the traditional community leaders were invited to a meeting at which the rationale, objectives, implementation procedures and

expectations of the project were presented prior to intervention implementation and discussed. The leaders understood the project, and each made a commitment to facilitate the project's activities in their respective communities. They contributed to the identification of community volunteers to be involved in project activities in their communities. These included persons able to speak local (Kapsiki) and an official language (English or French) living in one of targeted communities. These community volunteers were trained to visit households, introduce the project to the head of household, and seek his permission to invite members of their household to visit the project site as potential participants. The community volunteers were also involved in reminding the people included in the study of their visit schedules.

Enrollment. Households in the Mogode health area in the Mogode health district were visited by community volunteers to introduce the project to household heads and obtain permission to brief household inhabitants and invite those eligible to the study site prepared for this purpose with the collaboration of the Mogode district hospital. Participants presenting themselves at the study site were informed about the study. Those who consented were screened for eligibility and selection criteria. Eligible participants meeting the inclusion criteria were assigned to the two arms of the study by randomization. During the follow-up period, participants were reminded by telephone or through home visits of the dates and times of their follow-up visits. Enrollment of participants for the present trial was conducted from February to June 2021.

Determining participants age and prior vaccination status. For eligible participants, the age was collected on the identity card or birth certificate. In the absence of these documents, age was tracked using the documented ages of other children in the household or neighborhood, or using major events whose timing in the past was known. Vaccination status 4 years prior to OCV was determined using the vaccination record, or tracked using photos of the vaccine, the vaccine administration protocol and specific questions asked to participants or guardians (the period of the campaign, the presence of the participant in the community during the campaign, the description of the vaccine vial and the taste of the vaccine if possible).

Randomization. Randomization was stratified by age group and prior exposure status to OCV. Thus, in the stratum of each age group, participants who had received zero, one or two doses of OCV were randomly assigned in blocks of two to Arm A or Arm B. The team responsible for randomization was different from the team enrolling and randomly assigning participants to study arms. The randomization procedure provided to generate the four possible combinations of blocks of two (AA, AB, BA, BB, each combination indicating the assignment of each pair of participants to the two study arms) and ranked in each stratum of of expected participants in random order and associated with numbers to generate unique code that indicted the study arm of each participant. The code was printed on a label, inserted into opaque envelopes arranged per stratum in order and numbered. The randomization was implemented by taking par stratum (age group and vaccination status) an envelope for an included patient following the order of numbers dictated by the randomization team, opening it, removing the label and assigning the number to the participant.

Interventions

The administration of the vaccine was conducted at the study implementation site. Participants in arm A received oral administration of one dose of OCV (Shanchol, Lots 1: SCN006A19 and Lot 2: SCN034A20) on day zero. Participants in arm B received two doses of OCV (same batch); the first dose was on day 0 and the second dose was on day 14 to 16. Shanchol consists of a mixture of killed *V. cholerae* O1 and O139 in single-dose glass vial containing 1.5 mL of vaccine. The entire vial of OCV was administered orally to each participant irrespective of their age. The vaccine vial was shaken to ensure mixing and was inspected by the study nurse before administration. To monitor for safety, all participants were observed for 30 minutes after taking the vaccine and had to report any adverse events occurring within two weeks of receiving the vaccine to the community worker or during the next study planned visit. For ethical reasons, and to ensure that all study participants had an opportunity to receive the recommended two doses of vaccine, the participants in arm A were invited to take the second OCV dose after their follow-up period, which ended on day 28, but no additional serum samples were collected to assess the serological response after this second dose in arm A.

Outcome assessment

In arm A, participants were followed up for 28 days with five blood draws and in arm B, for 42 days with six blood draws. The timing of the follow-up vaccinations and blood draws along with the acceptable visit windows for each event are shown in figure 1. The primary outcome was vibriocidal GMT and seroconversion rate measured four to six days following single dose OCV to assess and compare early immune responses between participants exposed to two doses OCV four years earlier to those exposed to zero doses four years earlier. Secondary outcomes included 1) Age group specific (1-4, 5-14, ≥15 years) and overall vibriocidal GMT and seroconversion rates measured at days 4-6, 9-12, 14-16 and 28-30 following OCV administration to compare vibriocidal responses between participants who received one or two OCV doses four years earlier and those who received zero doses four years earlier. 2) Vibriocidal GMT and seroconversion rates measured four days following a single OCV dose to compare early immune response between participants exposed to one or two doses of OCV four years earlier and those exposed to zero doses. 3) Vibriocidal GMT and seroconversion rate 14-16 days following exposure to the single dose (arms A and B) and days 18-20, 23-25, 28-30 and 42-44 for arm B.

Figure 1: Visit windows, vaccination and blood draw schedules

Laboratory procedures

The timing of the blood samples is shown in figure 2. Blood sample collection procedures depended on age group. For age group <5years, a fingerstick sample was obtained using a lancet and capillary tube to collect 350 µl of blood which was then diluted in 1400 µl normal saline which resulted in a 1:5 dilution of whole blood. For subjects ≥5 years 5 ml of venous blood was collected using a syringe. The blood samples were centrifuged and the serum was stored at -20°C until assayed.

For the vibriocidal assay, we used *V. cholerae* strains isolated from Cameroon (Inaba 600040 and Ogawa CPC-BACT-01) as previously described (19). The strains were incubated with serially diluted, heat-inactivated serum with exogenous guinea pig complement (Sigma Aldrich, catalogue # S234395) on a shaker (50 rev/min) at 37°C for 1 hour.[16] The starting dilution for the previously undiluted serum

was 1:10 while the previously diluted serum from young children was used without further dilution considering that the 1:5 dilution of whole blood to be equivalent to the 1:10 dilution of serum. Vibriocidal titers were defined as the reciprocal of the highest serum dilution which results in a 50% reduction in optical density (595 nm) compared with growth control without serum. Standard sera, comprising of high titer human serum and high titer rabbit serum were employed to normalize the outcomes and mitigate the effects of inter-experimental variation. Samples were tested in duplicates. Titers lower than the lowest detectable titer were assigned to have a titer equivalent to half of the lowest detectable titer. The threshold for inter- and intra-experimental variation was set at 2-fold. Seroconversion was defined as ≥ 4-fold rise in vibriocidal titers compared with baseline titer.

Data analysis

To identify confounders between OCV exposure status and between study arms we compared the distribution of participants' baseline characteristics between those exposed to zero OCV doses and those exposed to two doses four years earlier, and between those assigned to the two arms.

For the primary analysis, we compared the vibriocidal GMT and seroconversion rates on day 4-6 following the administration of a single dose of OCV between participants exposed to two OCV doses in 2017 and those exposed to zero doses using Student's T test and Z test respectively. For the estimate of vibriocidal seroconversion rate, logistic regression model was used for the adjustment using individual and household characteristics as adjustment variables.

For the secondary analyses, we carried out the same analysis per age group between participants exposed to 1 or 2 OCV doses and those exposed to zero doses in 2017. These estimates were also conducted on day 9-12, 14-16, and 28-30 following the single OCV dose administration for participants of arm A. Diagrams showing estimated of vibriocidal GMTs with a 95% confidence interval on the planned blood draw days flowing single dose OCV administration (Arm A) and two doses OCV administration (arm B) was generated for overall, by age group and according to 2017 OCV vaccination status. The comparison of vibriocidal GMT was done using the Student T test and

that of seroconversion rate using the Z test. Regarding the seroconversion rate. For the estimate of vibriocidal seroconversion rate, logistic regression model was used for the adjustment using individual and household characteristics as adjustment variables.

The comparison was made with one tailed 95% confidence interval. Analyses were conducted using Stata (Version 18).

Ethical Considerations

Permission from adequately informed community authorities was obtained before accessing eligible participants in households. All study participants were fully informed in an appropriate language about study justifications, objectives, procedures, implications, and expected results and were given the time to ask questions before deciding to consent or not to participate. Only those that provided their written informed consent were included. For minor participants (<21 years), parental permission was obtained. For minors aged between 12 and 20, their assent was sought and obtained prior to their inclusion in the study. Their assent was accompanied by a parental consent. Measures were taken to ensure confidentiality of patient data during inclusion, administration of interventions, patient sampling, data processing and analysis. All participants were observed 30 minutes after vaccine administration and visited to detect, report and investigate any adverse events occurring after vaccine administration. Similarly, measures were taken regarding the risk of infection, trauma and pain resulting from biological sampling. The research protocol was approved the Cameroon National Ethics committee for Human Health Research (N°: 2020/12/1321/CE/CNERCH/SP).

Results

Enrollment

The consort flow diagram for the study is shown in Figure 2. Of the 430 persons who were received in the study site, 3 did not consent to be screened and 77 did not meet inclusion criteria. 350 (81.4%)

participants were enrolled in the study with 136, 101 and 113 with OCV vaccination status four years earlier of 0, 1 and 2 doses respectively. For those who were included, 174 and 176 were randomly assigned to Arm A and Arm B respectively.

Figure 2: Consort flow diagram

Baseline characteristics of participants regarding study arms.

Table 1 presents the comparison of participants' characteristics per study arm. No difference in the distribution of these characteristics was detected.

Table 1: Distribution and comparison of participants characteristics per study arm

**Characteristics**	**Arm A (N=174)**	**Arm B (N=176)**
Sex, n(%)		
Male	72 (41.4)	69 (39.2)
Age group,n(%)		
1-4	37 (21.3)	37 (21.0)
5-14	69 (39.7)	69 (39.2)
≥15	68 (39.1)	70 (39.8)
Age median(IQR)		
	11 (23)	12 (25.5)
Floor material participants houses, n (%)		
Cement / Brick/Tiles	50 (28.7)	40 (22.7)
Using improved toilet facility, n (%)		
	19 (10.9)	15 (8.5)
Weight in kg, mean [CI]		
	34.4 [31.5, 37.4]	36 [33, 38.9]
Body Mass Index, mean [CI]		

	16.8[16.2,17.4]	16.9[16.3,17.6]
Baseline LVGMT Inaba, geometric mean [CI]		
	1[0.9,1.1]	1 [1,1.1]
Baseline LVGMT Ogawa, geometric mean [CI]		
	1.5[1.4,1.6]	1.4[1.3,1.5]
Vaccination status, n(%)		
0 dose	68 (39.1)	68 (38.6)
1 dose	49 (28.7)	52 (29.6)
2 doses	57 (32.8)	56 (31.8)

LVGMT= log of vibriocidal Geometric mean titer, VSCR= vibriocidal seroconversion rate.

Comparison of characteristics of participants per OCV exposure status, four years earlier.

Table 2 shows a comparison of the distribution of the characteristics of participants exposed to two or at least one dose of OCV compared with those exposed to zero dose. The comparison reveals relative differences in distribution for age groups, housing materials between those who did not receive OCV earlier and those who did.

Table 2: Baseline comparison of participants who received no OCV earlier and those who received one or two doses earlier.

**Characteristics**	**Received zero doses earlier compared to those who received two doses earlier**		**Zero doses compared to those who received one or two doses earlier**
	**Zero dose (N=136)**	**Two doses(N=113)**	**One or two doses (N=214)**
Sex, n(%)			
Male	53 (39.0)	48 (42.5)	88 (41.1)
Age group n (%)			

1-4	44(32.4)	21 (18.6)	30 (14.0)
5-14	46 (33.8)	46 (40.7)	92 (43.0)
≥15	46 (33.8)	46 (40.7)	92 (43.0)
Age median(IQR)			
	9 (20.0)	12 (23.0)	13 (29.0)
Housing material, n (%)			
Cement / Brick/Tiles	47 (34.6)	17 (15.0)	43 (20.1)
Using improved toilet facility n(%)			
Yes	18 (13.2)	9 (8.0)	16 (7.5)
Weight in kg, mean [CI]			
	31.9[28.6,35.3]	36[32.3, 39.7]	37.3 [34.6, 39.9]
Body Mass Index, mean [CI]			
	16.4[15.7,17.1]	17[16.2,17.9]	17.2[16.6,17.8]
Baseline VGMT Inaba , [CI]			
LVGMT Inaba	1[0.9,1.1]	1 [0.9,1.1]	1 [1,1.1]
VGMT Inaba	9.3[7.6,11.5]	9.8 [7.9,12.2]	11.2 [9.3,13.4]
Baseline LVGMT Ogawa , [CI]			
LVGMT Ogawa	1.3[1.2, 1.5]	1.5 [1.4, 1.7]	1.5[1.4, 1.6]
VGMT Ogawa	21.2[15.8, 28.3]	34.5 [24.6, 48.5]	34.5[27.0, 44.0]

VGMT= vibriocidal Geometric mean titer

Comparison of the early (day 4-6) vibriocidal response in participants who received zero, one or two doses of OCV four years earlier.

The early immune responses (vibriocidal GMT and seroconversion rate) of participants (all age group, per age group and for serotypes Inaba and Ogawa) exposed to two or at least one dose of OCV versus those exposed to zero dose is presented in Table 3. Early rise in vibriocidal titers was rare in participants exposed four years earlier to two OCV doses, one OCV dose and no difference was observed when compared to those who were not reported to be administered an OCV vaccine dose in 2017. This was consistent across age groups and serotypes (Ogawa and Inaba).

Table 3: Comparison of Vibriocidal GMT and Seroconversion rates 4-6 days after a single dose of OCV depending on receipt of OCV four years earlier.

**Age group and outcome (VGMT and VSCR)**	**Early vibriocidal titer changes among those who received zero doses of OCV and those who received two doses earlier (Arm A)**			**Early vibriocidal titer changes among those who received zero doses of OCV and those who received one or two doses earlier (Arm A)**	
	**Zero dose**	**Two doses**	**P value (adjusted)**	**One or two doses**	**P value (adjusted)**
**All age group**	**N=59**	**N=49**		**N=92**	
**LVGMT Inaba,[CI]+**	1.2 [1;1.4]	1.2 [1;1.4]	0.7	1.1 [1;1.3]	0.8
**LVGMT Ogawa,[CI]+**	1.5 [1.3;1.7]	1.7 [1.5;1.9]	0.1	1.6 [1.5;1.8]	0.2
**VSCR Inaba, %[CI]***	23.7 [12.5;34.9]	22.4 [10.3;34.6]	0.9 (0.60)	12 [5.2;18.7]	0.1 (0.1)
**VSCR Ogawa, %[CI]***	15.3 [5.8;24.7]	18.4 [7.1;29.6]	0.70 (0.5)	10.9 [4.4;17.4]	0.4 (.60)

**1-4 years**	**N=17**	**N=9**		**N=13**	
**LVGMT Inaba,[CI]+**	1.2 [0.9;1.6]	1.3 [0.7;1.9]	0.6	1.1 [0.7;1.5]	1
**LVGMT Ogawa,[CI]+**	1.2 [0.8;1.5]	1.5 [1;2]	0.2	1.4 [.9;1.8]	0.4
**VSCR Inaba, % [CI]***	29.4 [5.3;53.6]	33.3 [0;71.8]	0.8(0.9)	23.1 [5.3;49.6]	0.70 (0.70)
**VSCR Ogawa, % [CI]***	29.4 [5.3;53.6]	44.4 [3.9;85]	0.4(.3)	30.8 [5.3;59.8]	0.9 (0.60)
**5-14 years**	**N=22**	**N=22**		**N=42**	
**LVGMT Inaba,[CI]+**	1.2 [.8;1.5]	1 [0.8;1.2]	0.3	1 [0.8;1.2]	0.3
**LVGMT Ogawa,[CI]+**	1.4 [1.2;1.6]	1.5 [1.2;1.8]	0.9	1.5 [1.3;1.7]	0.6
**VSCR Inaba, % [CI]***	22.7 [3.7;41.7]	18.2 [0.7;35.7]	0.70(0.3)	9.5 [3.7;18.8]	0.1 (0)
**VSCR Ogawa, % [CI]***	18.2 [0.7;35.7]	13.6 [0;29.2]	0.70(.2)	7.1 [.70;15.3]	0.2 (0)
**> 14 years**	**N=20**	**N=18**		**N=37**	
**LVGMT Inaba,[CI]+**	1.2 [.8;1.5]	1.4 [1.1;1.8]	0.2	1.3 [1.1;1.6]	0.4
**LVGMT Ogawa,[CI]+**	1.8 [1.4;2.2]	2.1 [1.7;2.5]	0.3	1.8 [1.5;2.1]	0.9

**VSCR Inaba, % [CI]***	20 [.8;39.2]	22.2 [0.9;43.5]	0.9(0.2)	10.8 [0.8;21.3]	0.3 (1)
**VSCR Ogawa, % [CI]***	0 [0;0]	11.1 [0;27.2]	0.1	8.1 [0;17.3]	0.2
+ P.value are from Wilcoxon test, * P.value are from chi-square, LVGMT= log of vibriocidal Geometric mean titer, VSCR= vibriocidal seroconversion rate. The logistic regression model has been used for the adjustment with seroconversion status as dependent variable, vaccination status in 2017 as main independent variable and other variables (Sex, Age group, floor material, using improved toilet facility, weight, and height) as adjustment variables (age group was considered as adjustment variable for all age).					

Distribution and comparison of vibriocidal GMT and seroconversion rates 9-12 days after a single OCV dose depending on receipt of OCV four years earlier

Table 4 presents the comparison of vibriocidal GMT and seroconversion rate 9-12 days following single OCV dose depending on receipt of OCV earlier. The Ogawa vibriocidal GMT at day 9-12 following single OCV dose administration in all age group, and each age group significantly increased among people exposed four years earlier to zero, two doses and one or two OCV doses and was significantly higher in those exposed to one or two doses OCV. The similar tendency was observed for Inaba serotype but there was no difference comparing vibriocidal GMT in those exposed four years earlier to OCV to those not exposed. The increasing of vibriocidal seroconversion rate for Ogawa and Inaba was also observed but no difference was detected between those exposed to OCV four years and those not exposed.

Table 4: Comparison of Vibriocidal GMT and Seroconversion rates 9-11 days after a single dose of OCV depending on receipt of OCV four years earlier.

Blood characteristics (outcomes) 9-11 days	Arm A Number of OCV doses exposed four year earlier (zero dose and two doses)	Arm A Number of OCV doses exposed four year earlier (zero dose and one or two doses)

after the single dose administration	Zero dose	Two doses	P-value (adjusted)	One or two doses	P-value (adjusted)
All age group	N=59	N=47		N=87	
LVGMT Inaba,[CI]+	1.8 [1.6;2]	1.9 [1.7;2.2]	0.5	1.9 [1.7;2.1]	0.7
LVGMT Ogawa,[CI]+	1.9 [1.8;2.1]	2.6 [2.4;2.8]	0	2.4 [2.2;2.5]	0
VSCR Inaba, %[CI]*	62.7 [50;75.4]	68.1 [54.3;81.9]	0.60 (0.4)	64.4 [54.1;74.6]	0.8 (0.60)
VSCR Ogawa, %[CI]*	61 [48.2;73.8]	70.2 [56.6;83.8]	0.3 (0.2)	65.5 [55.3;75.7]	0.60 (0.3)
1-4 years	N=19	N=8		N=11	
LVGMT Inaba,[CI]+	2 [1.6;2.4]	2.5 [1.8;3.2]	0.2	2.2 [1.6;2.8]	0.6
LVGMT Ogawa,[CI]+	1.8 [1.4;2.2]	2.6 [2;3.1]	0	2.4 [1.9;2.9]	0.1
VSCR Inaba, % [CI]*	73.7 [51.9;95.5]	100 [100;100]	0.1	90.9 [51.9;111.2]	0.3 (1)
VSCR Ogawa, % [CI]*	73.7 [51.9;95.5]	100 [100;100]	0.1	90.9 [51.9;111.2]	0.3 (.60)
5-14 years	N=20	N=21		N=42	
LVGMT Inaba,[CI]+	1.8 [1.5;2.1]	1.9 [1.5;2.3]	0.8	1.9 [1.7;2.2]	0.6
LVGMT Ogawa,[CI]+	2.1 [1.9;2.3]	2.5 [2.2;2.9]	0	2.4 [2.2;2.6]	0
VSCR Inaba, % [CI]*	60 [36.5;83.5]	71.4 [50.4;92.5]	0.4(1)	73.8 [36.5;87.7]	0.3 (0.70)
VSCR Ogawa, % [CI]*	80 [60.8;99.2]	71.4 [50.4;92.5]	0.5(0.5)	66.7 [60.8;81.5]	0.3 (0.3)
> 14 years	N=20	N=18		N=34	
LVGMT Inaba,[CI]+	1.6 [1.3;2]	1.8 [1.3;2.2]	0.7	1.7 [1.4;2]	0.7
LVGMT Ogawa,[CI]+	1.9 [1.6;2.3]	2.7 [2.3;3]	0	2.4 [2.1;2.7]	0.1

**VSCR Inaba, % [CI]***	55 [31.1;78.9]	50 [24.4;75.6]	0.8(0.70)	44.1 [31.1;61.7]	0.4 (0.5)
**VSCR Ogawa, % [CI]***	30 [8;52]	55.6 [30.1;81]	0.1(0.3)	55.9 [8;73.5]	0.1 (0.1)

+ P.value from Wilcoxon test, * P.value from chi-square, LVGMT= Log of vibriocidal Geometric mean titer, VSCR= vibriocidal seroconversion rate The logistic regression model has been used for the adjustment with seroconversion status as dependent variable , vaccination status in 2017 as main independent variable and other variables (Sex, Age group, floor material, using improved toilet facility ,weight, and height) as adjustment variables (age group has been included only at the global level).

Trends of vibriocidal GMT following single dose administration of OCV

Figures 4-a and 4-b show that for all and each age groups, the vibriocidal GMT for Inaba serotype at days 9-12, 14-14 and 28-30 days following single OCV dose administration was not higher in participants exposed to two OCV doses, one or two OVC doses four years earlier than those who did received OCV at that period. The vibriocidal GMT for Ogawa serotype at days 9-12, 14-14 and 28-30 days following single OCV dose administration was higher in participants exposed to two OCV doses, one or two OCV doses four years earlier than those who did received OCV at that period. This tendency was observed for all and each age group.

Figure 3-a: Vibriocidal Geometric Mean Titer following single OCV of the three participating OCV status four year earlier through the span of the study.

Figure 3-b: Age stratified vibriocidal Geometric Mean Titer following single dose administration of the three participants OCV status four year earlier through the span of the study.

Trends of vibriocidal GMT and seroconversion in study arms A and B

The GMT and the two study arms are shown in Figure 4. For both Ogawa and Inaba serotypes, for all ages and for each age group and whether the subject had received OCV earlier, the administration of the second dose of OCV (arm B) did not lead to an increase in GMT vibriocidal values. No difference was detected between vibriocidal GMT titre of arm A compared to arm B in participants exposed to one or two OCV doses four years earlier; as was the case in those who had not been vaccinated four years earlier.

Figure 4-a: Vibriocidal GMT of the participants who received 1 or 2 OCV doses earlier in Arms A and B through the span of the study.

Figure 4-b: Age stratified Vibriocidal GMT of the participants who received 1 or 2 OCV doses earlier in Arms A and B through the span of the study.

Comparison of Vibriocidal GMT and Seroconversion rates 28-30 days between arm A and arm B.

Table 5 presents the comparison between arm A and arm B of Vibriocidal GMT and Seroconversion rates 28-30 days from day 0. There was no difference between arms A and B for all and each age group or serotype.

Table 5: Comparison of Vibriocidal GMT and Seroconversion rates 28-30 days after the first dose of OCV arm A and arm B.

**Participants**	**Arm A and arm B immune response 28-30 days after the first dose administration**		
	**Arm A**	**Arm B**	**P-value (adjusted)**
**All age group**	N=147	N=150	
**LVGMT Inaba,[CI]+**	1.5 [1.4;1.6]	1.6 [1.5;1.8]	0.2
**LVGMT Ogawa,[CI]+**	1.9 [1.7;2]	1.9 [1.8;2]	0.6
**VSCR Inaba, %[CI]***	49.7 [41.5;57.8]	50.3 [42.2;58.5]	0.9 (0.8)
**VSCR Ogawa, %[CI]***	33.3 [25.6;41]	43.3 [35.3;51.4]	0.1 (0.1)

**1-4 years**	N=31	N=33	
**LVGMT Inaba,[CI]+**	1.4 [1.2;1.7]	1.3 [1.1;1.5]	0.5
**LVGMT Ogawa,[CI]+**	1.4 [1.2;1.7]	1.4 [1.2;1.6]	1
**VSCR Inaba, % [CI]***	45.2 [26.6;63.7]	31.3 [14.3;48.2]	0.3 (0.2)
**VSCR Ogawa, % [CI]***	38.7 [20.5;56.9]	45.5 [27.5;63.4]	0.60 (0.60)
**5-14 years**	N=61	N=63	
**LVGMT Inaba,[CI]+**	1.5 [1.3;1.7]	1.9 [1.7;2.1]	0
**LVGMT Ogawa,[CI]+**	1.9 [1.8;2.1]	2.1 [2;2.3]	0.1
**VSCR Inaba, % [CI]***	50.8 [37.9;63.7]	63.5 [51.3;75.7]	0.2 (0.1)
**VSCR Ogawa, % [CI]***	41 [28.3;53.7]	50.8 [38.1;63.5]	0.3 (0.2)
**> 14 years**	N=55	N=54	
**LVGMT Inaba,[CI]+**	1.6 [1.4;1.8]	1.6 [1.4;1.8]	1
**LVGMT Ogawa,[CI]+**	2 [1.8;2.2]	1.9 [1.7;2.1]	0.7
**VSCR Inaba, % [CI]***	50.9 [37.3;64.5]	46.3 [32.6;60]	0.60 (0.60)
**VSCR Ogawa, % [CI]***	21.8 [10.6;33.1]	33.3 [20.3;46.3]	0.2 (0.1)

+ P-value from Wilcoxon test, * P-value from chi-square, LVGMT= Log of vibriocidal Geometric mean titer, VSCR= vibriocidal seroconversion rate. The logistic regression model has been used for the adjustment with seroconversion status as dependent variable, vaccination status in 2017 as main independent variable and other variables (Sex, Age group, floor material, using improved toilet facility, weight, and height) as adjustment variables (age group has been included only at the global level).

Detected adverse events following OCV administration.

Five adverse events were detected including 2 cases of nausea in the 30 minutes following immunization and 03 cases of diarrhea in the fourteen days following OCV administration. None of these cases was serious.

Discussion

The present study was intended to identify a rapid response to a dose of OCV if the subject had received the OCV vaccine earlier indicating that these people had responded with a booster response. However, we did not observe rapid responses in those who had received OCV earlier. In fact, an early response was seen rarely in any of the groups and there was not a significant difference in the proportion with a rapid response between those who had received OCV earlier and those who did not. This finding was consistent for each of age group (1-4, 5-14, ≥15 years old) and for both Ogawa and Inaba vibriocidal assays.

We did observe a higher peak Ogawa vibriocidal GMT after the initial dose OCV but not a higher Inaba GMT in those who had received OCV earlier compared to those who had not received OCV earlier. This was observed both for the entire age group and for age specific groups.

In Arm B, there were no rapid responses on day 18 (4 to 6 days after dose 2) to suggest a booster response to this second dose. The GMT at day 14, 28-days following administration of one dose of OCV in arm A and two doses 14 days apart in arm B did not differ for overall participants, for each age group, serotype Ogawa and Inaba and by prior exposure to OCV vaccine status.

The administration of two doses Oral Cholera Vaccine (OCV) is known to protect about 65% and a lower percentage of children aged 1-4 years for five years following the vaccination (12). The present study was planned with the hypothesis that the magnitude and speed of the immune response following OCV vaccination would correlate with immune memory and would therefore be higher and faster in previously vaccinated individuals than in those not previously vaccinated. The vibriocidal GMT and seroconversion rate for the Ogawa and Inaba serotypes was not higher for overall participants nor for each of the three age groups among those exposed four years earlier to one or two doses

compared to those exposed to zero OCV dose four years earlier. These results are consistent with those of a study conducted in Bangladesh measuring immune response in all age groups on day 3 following administration of a single dose of Shanchol vaccine (13). The present study is the first study to examine these responses in these age groups, <u><</u>5 years, 5-14 and ≥ 15 years) in people who had received two doses of OCV vaccine 4 years earlier. It is unlikely that not detecting differences of GMT and response rates between participants exposed to one or two doses of OCV and those exposed to zero doses be explained by differences in the distribution of some characteristics between exposure status at baseline, since these differences were taken into account in the adjustment of the estimation of the comparison tests. Similarly, it is unlikely that repeated, low-grade or undetected natural exposure to Vibrio cholerae in the study setting have reduced the differences in vibriocidal antibody responses between vaccinated and unvaccinated participants. Following the cholera vaccination campaign organised in 2017, our team conducted a year-long epidemiological surveillance of cholera, screening suspected cases using rapid diagnostic tests for cholera. During this surveillance period, no positive case was detected.

A number of assumptions that may explain the lack of difference in early immune response according to the status of OCV vaccination status deserve to be reviewed. The relatively high frequency of malnutrition in the Far North Cameroun region, as documented by recent demographic health surveys, could explain the low immune response in people who have been previously vaccinated, but in this case, a difference in immune response in adults would have been observed (20). The vaccination status of most of the participants was not based on vaccination cards as these cards were no longer available but determined using process inspired from a previous study that was documented reliable in assessing immunization status in people vaccinated with no evidence (21). Despite the fact that the association between vibriocidal antibody titers and cholera immunity is not absolute, the present result suggests that early immune response resulting from single OCV administration is not sufficient to ensure cholera protection in all age groups (22). This trend should be confirmed exploring in the same context the use of other cholera vaccine immunity markers which appear in studies conducted

in other contexts to show a higher booster immune response in populations previously exposed to vaccination (13).

The immune response following administration of a dose of OCV at 9-11 days after administration was higher for serotype Ogawa in each of the age groups of individuals previously exposed to cholera vaccination and was not higher for Inaba. The difference in kinetics of Inaba and Ogawa serotypes may be related to the earlier exposure the Ogawa serotype that was identified during the 2017 outbreak.(23–25). The immune response curve following the first dose of OCV vaccine shows an increasing vibriocidal concentration, peaking at 9-11 days after a single dose OCV administration and then gradually decreasing. The curve following the second dose peaks days 9-11 and days 23-25 but on days 28-30, the immune response among those previously exposed to OCV was not different among those exposed to 1 dose OCV compared to those exposed to two doses. To the best of our knowledge, no study describing the booster immune response curve following the second booster dose has been published, nor has a study reported the booster immune response following the first and second booster doses of OCV in the same population. The data collected in our study do not allow to explain the absence of difference in booster immune response at 28-day following one and two doses of OCV vaccination. This lack of difference may be explained by a local intestinal immune response that is probably triggered by the OCV first dose that partially neutralizes the second dose to reduce its bioavailability and the resulting immune response (26). The consistency of the observed findings needs to be explored in different setting and over other biomarkers to guide the planning of revaccination populations in need.

In summary, we had hoped to observe a rapid vibriocidal response to participants who had been vaccinated four years earlier. We also intended to determine if the magnitude of the vibriocidal response was of higher in those vaccinated earlier. However, we did not observe a rapid vibriocidal response in any of the groups and there was no difference in this response depending on earlier vaccination. Similarly, except for a higher Ogawa in those vaccinated earlier, the vibriocidal titers were similar in the different groups. The study does have implications for determining the number of doses

of vaccine to be delivered when revaccinating populations that were vaccinated earlier. Since the previously vaccinated people responded in the same way as those not previously vaccinated, we feel this data suggests that that re-vaccination of previously vaccinated groups should follow the same recommendations as vaccination of groups not previously vaccinated; that is, that two doses is appropriate whenever this is feasible.

The study presented some limitations. The study took place at the start of the COVID-19 pandemic in Cameroon, which led to delays in the supply of vaccines, reducing the enrolment window for eligible participants in the 1-5 age group in 2017. This resulted in the sample size for this age group not being reached. Also, data on the nutritional status of participants that could have contributed in guiding the understanding of the immune response in the study arms were not collected.

Acknowledgements. We acknowledge and thank the participants in Mogode who participated in this research study.

Potential conflicts of interest. All authors: No reported conflicts of interest.

Data availability: Data presented and analyzed for the present manuscript are available as "Supplementary information file 1"

Funding:

This work was supported by a grant from the Wellcome Trust to JA (grant number: 215663/Z/19/Z) and a grant from the National Institute of Allergy and Infectious Disease to DAS (5R01AI123422). The Wellcome Trust provided suggestions as to the study design, but neither funder had any role in data collection and analysis, decision to publish, or preparation of the manuscript.

References

1. Kanungo S, Azman AS, Ramamurthy T, Deen J, Dutta S. Cholera. The Lancet. 2022 Apr 9;399(10333):1429–40.

2. World Health Organization. Weekly Epidemiological Record, 2023, vol. 98, 38 [full issue]. Wkly Epidemiol Rec Relevé Épidémiologique Hebd. 2023 Sep 22;98(38):431–52.

3. World Health Organization. Weekly Epidemiological Record, 2024, vol. 99, 36 [full issue]. Wkly Epidemiol Rec Relevé Épidémiologique Hebd. 2024 Sep 6;99(36):479–96.

4. Pezzoli L. Global oral cholera vaccine use, 2013–2018. Vaccine. 2020 Feb 29;38:A132–40.

5. Shaikh H, Lynch J, Kim J, Excler JL. Current and future cholera vaccines. Vaccine. 2020 Feb 29;38:A118–26.

6. Wierzba TF. Oral cholera vaccines and their impact on the global burden of disease. Hum Vaccines Immunother. 2019;15(6):1294–301.

7. Song KR, Lim JK, Park SE, Saluja T, Cho SI, Wartel TA, et al. Oral Cholera Vaccine Efficacy and Effectiveness. Vaccines. 2021 Dec 15;9(12):1482.

8. Msyamboza KP, M'bang'ombe M, Hausi H, Chijuwa A, Nkukumila V, Kubwalo1 HW, et al. Feasibility and acceptability of oral cholera vaccine mass vaccination campaign in response to an outbreak and floods in Malawi. Pan Afr Med J [Internet]. 2016 Apr 20 [cited 2024 Oct 21];23(203). Available from: https://www.panafrican-med-journal.com//content/article/23/203/full

9. P S, Ck O, K R. A Quantitative Systematic Review and Meta-Analysis of the Effectiveness of Oral Cholera Vaccine as a Reactive Measure in Cholera Outbreaks. Prehospital Disaster Med [Internet]. 2018 Feb [cited 2024 Oct 21];33(1). Available from: https://pubmed.ncbi.nlm.nih.gov/29317005/

10. Wierzba TF. Oral cholera vaccines and their impact on the global burden of disease. Hum Vaccines Immunother. 2018 Oct 12;15(6):1294–301.

11. Amisu BO, Okesanya OJ, Adigun OA, Manirambona E, Ukoaka BM, Lawal OA, et al. Cholera resurgence in Africa: assessing progress, challenges, and public health response towards the 2030 global elimination target. Infez Med. 32(2):148–56.

12. World Health Organization. Cholera vaccine: WHO position paper, August 2017 – Recommendations. Vaccine. 2018 Jun 7;36(24):3418–20.

13. Chowdhury F, Bhuiyan TR, Akter A, Bhuiyan MS, Khan AI, Tauheed I, et al. Augmented immune responses to a booster dose of oral cholera vaccine in Bangladeshi children less than 5 years of age: Revaccination after an interval of over three years of primary vaccination with a single dose of vaccine. Vaccine. 2020 Feb 11;38(7):1753–61.

14. Kanungo S, Desai SN, Saha J, Nandy RK, Sinha A, Kim DR, et al. An Open Label Non-inferiority Trial Assessing Vibriocidal Response of a Killed Bivalent Oral Cholera Vaccine Regimen following a Five Year Interval in Kolkata, India. PLoS Negl Trop Dis. 2015 May 29;9(5):e0003809.

15. Rahman A, Rashu R, Bhuiyan TR, Chowdhury F, Khan AI, Islam K, et al. Antibody-Secreting Cell Responses after Vibrio cholerae O1 Infection and Oral Cholera Vaccination in Adults in Bangladesh. Clin Vaccine Immunol. 2013 Oct;20(10):1592–8.

16. Ritter AS, Chowdhury F, Franke MF, Becker RL, Bhuiyan TR, Khan AI, et al. Vibriocidal Titer and Protection From Cholera in Children. Open Forum Infect Dis. 2019 Apr 1;6(4):ofz057.

17. Bi Q, Ferreras E, Pezzoli L, Legros D, Ivers LC, Date K, et al. Protection against cholera from killed whole-cell oral cholera vaccines: a systematic review and meta-analysis. Lancet Infect Dis. 2017 Oct 1;17(10):1080–8.

18. Ateudjieu J, Yakum MN, Goura AP, Tembei Ayok M, Guenou E, Kangmo Sielinou CB, et al. An innovative approach in monitoring oral cholera vaccination campaign: integration of a between-round survey. BMC Public Health. 2022 Feb 5;22(1):238.

19. Ateudjieu J, Sack DA, Nafack SS, Xiao S, Tchio-Nighie KH, Tchokomeni H, et al. An Age-stratified, Randomized Immunogenicity Trial of Killed Oral Cholera Vaccine with Delayed Second Dose in Cameroon. Am J Trop Med Hyg. 2022 Nov 14;107(5):974–83.

20. Institut National de la Statistique (INS), ICF. Enquête Démographique et de Santé du Cameroun 2018 [Internet]. 2020. Available from: https://dhsprogram.com/pubs/pdf/FR360/FR360.pdf

21. Ateudjieu J, Tchio-Nighie KH, Yakum MN, Goura AP, Amada L, Sonkoua I, et al. Innovative approach in assessing the children's immunization status when it cannot be documented. J Public Health Afr. 2023 Sep 30;14(9):7.

22. Iyer AS, Harris JB. Correlates of Protection for Cholera. J Infect Dis. 2021 Dec 15;224(Supplement_7):S732–7.

23. Debes AK, Ateudjieu J, Guenou E, Lopez AL, Bugayong MP, Retiban PJ, et al. Evaluation in Cameroon of a Novel, Simplified Methodology to Assist Molecular Microbiological Analysis of V. cholerae in Resource-Limited Settings. PLoS Negl Trop Dis. 2016 Jan 6;10(1):e0004307.

24. Weil AA, Becker RL, Harris JB. Vibrio cholerae at the Intersection of Immunity and the Microbiome. mSphere. 2019 Nov 27;4(6):e00597.

25. Honda K, Takeda K. Regulatory mechanisms of immune responses to intestinal bacteria. Mucosal Immunol. 2009 May 1;2(3):187–96.

26. Liu Y, Zhou J, Yang Y, Chen X, Chen L, Wu Y. Intestinal Microbiota and Its Effect on Vaccine-Induced Immune Amplification and Tolerance. Vaccines. 2024 Aug;12(8):868.

Supporting information files legend

Supporting information file 1: Database

Supporting information file 2: Annex tables

Supporting information file 3: Ethical clearance

Supporting information file 4: Trial protocol

Supporting information file 5: Consort Checklist

Figure 1: Visit windows, vaccination and blood draw schedules

Click here to access/download;Figure;Figure 2.png

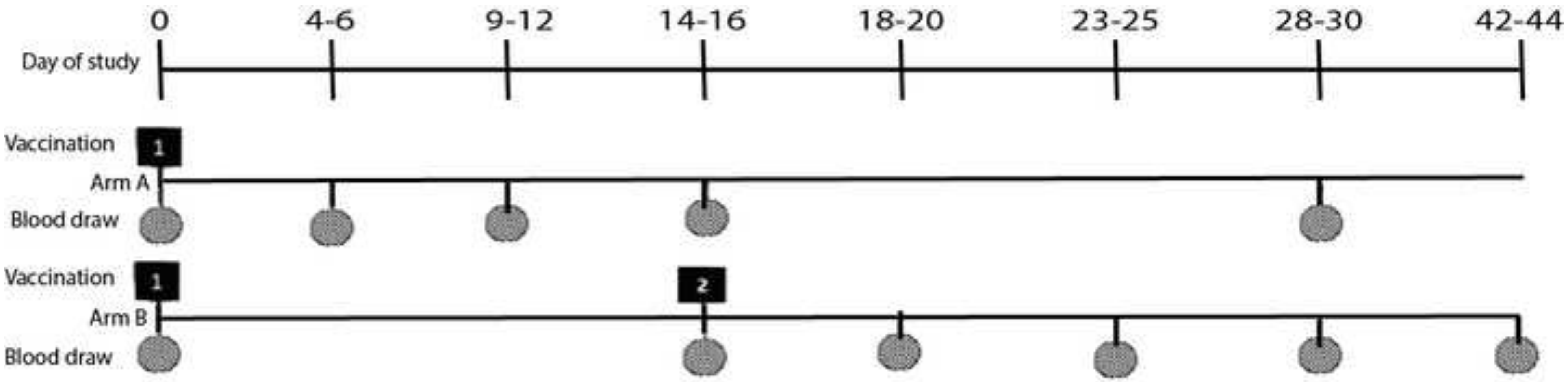

Figure 4-b: Age stratified Vibriocidal GMT of the participants who received 1 or 2 OCV doses earlier in Arms A and B through the span of the study

Click here to access/download;Figure;Figure 5b.tif

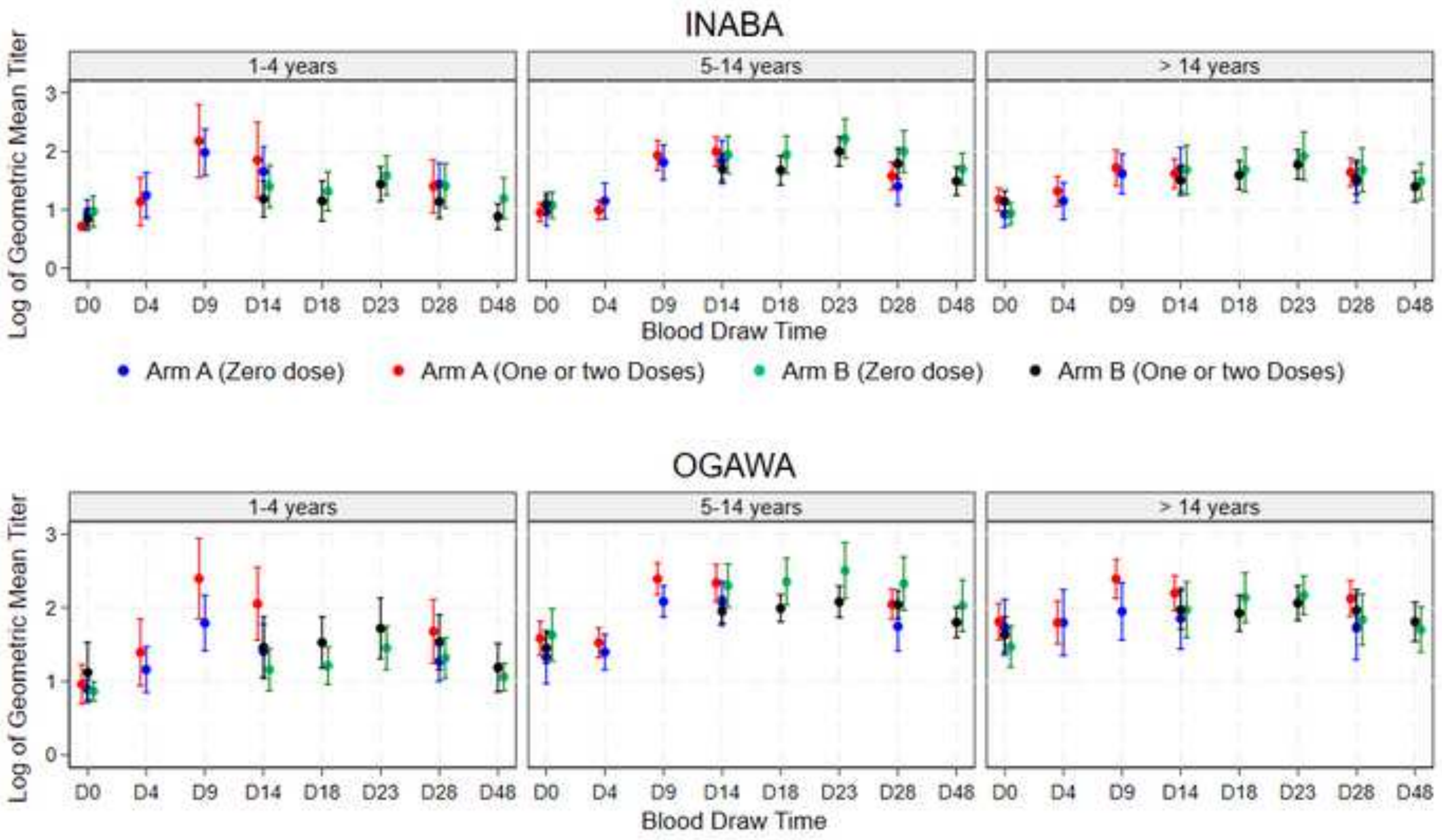

Figure 2: Consort Flow Diagram

Click here to access/download;Figure;Consort Flow Chart.pdf

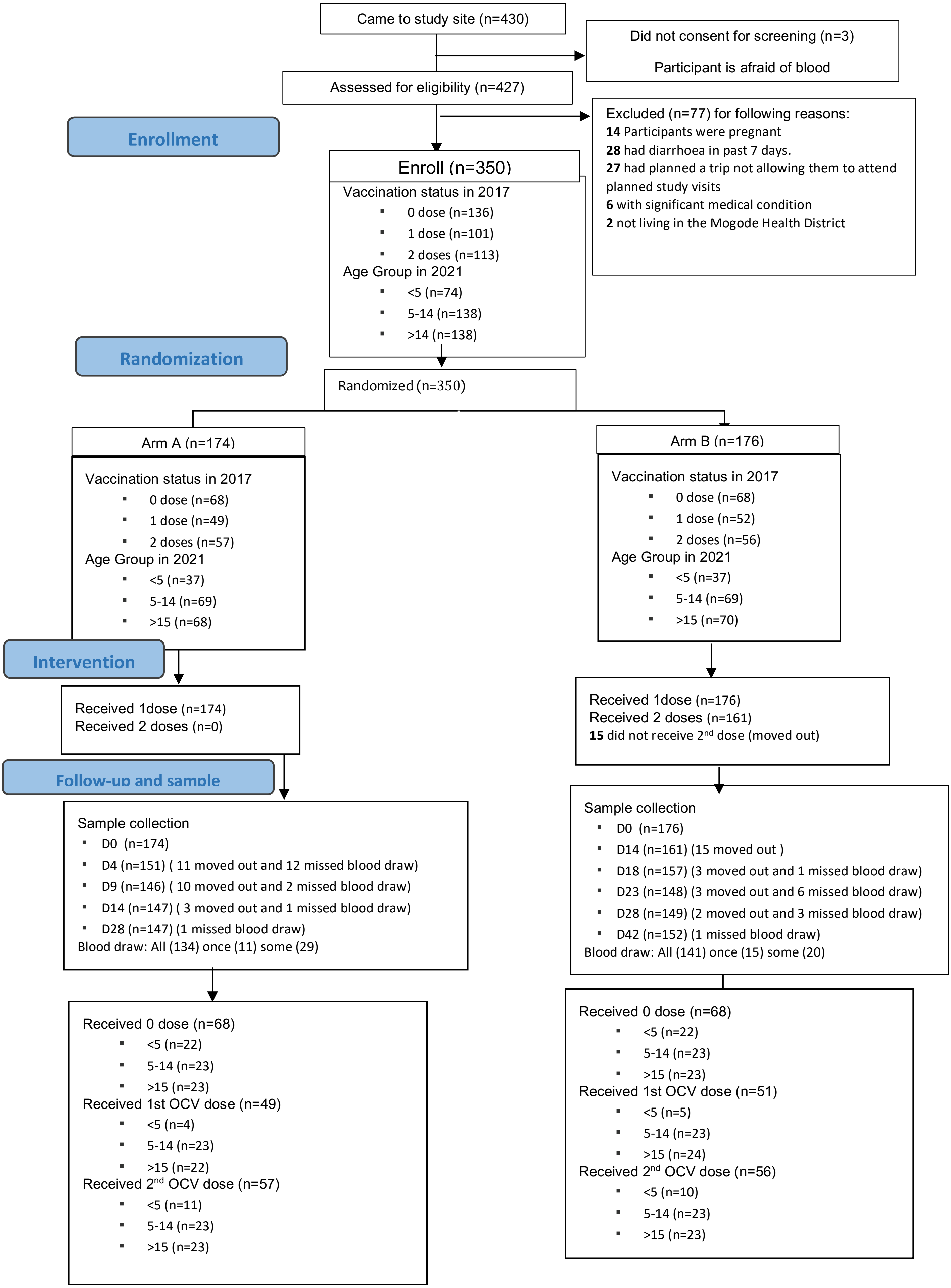

Figure 3-a: Vibriocidal Geometric Mean Titer following single OCV of the three participating OCV status four year earlier through the span of the study

Click here to access/download;Figure;Figure 4a.tif

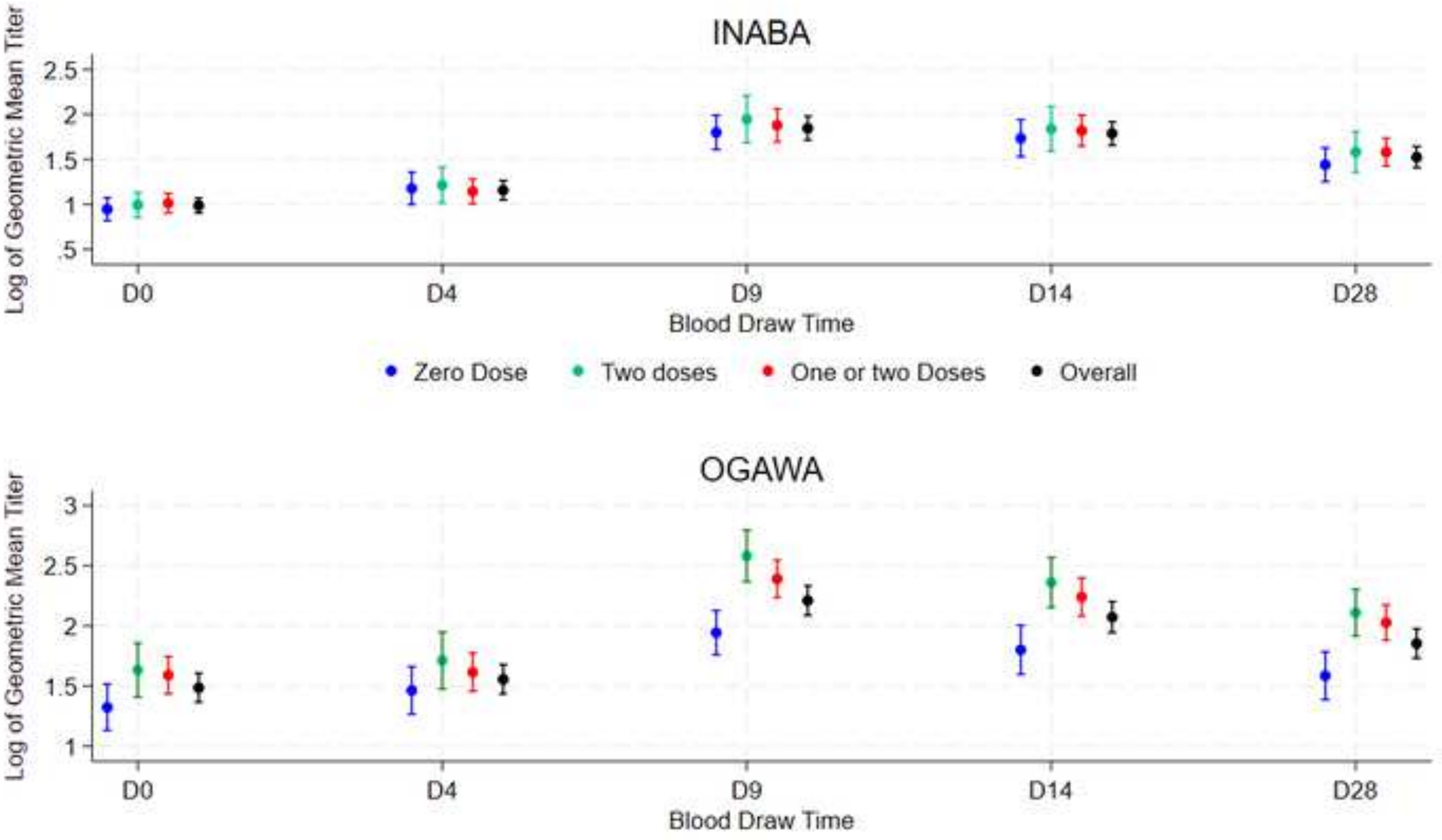

Figure 3-b: Age stratified vibriocidal Geometric Mean Titer following single dose administration of the three participants OCV status four year earlier through the span of

Click here to access/download;Figure;Figure 4b.tif

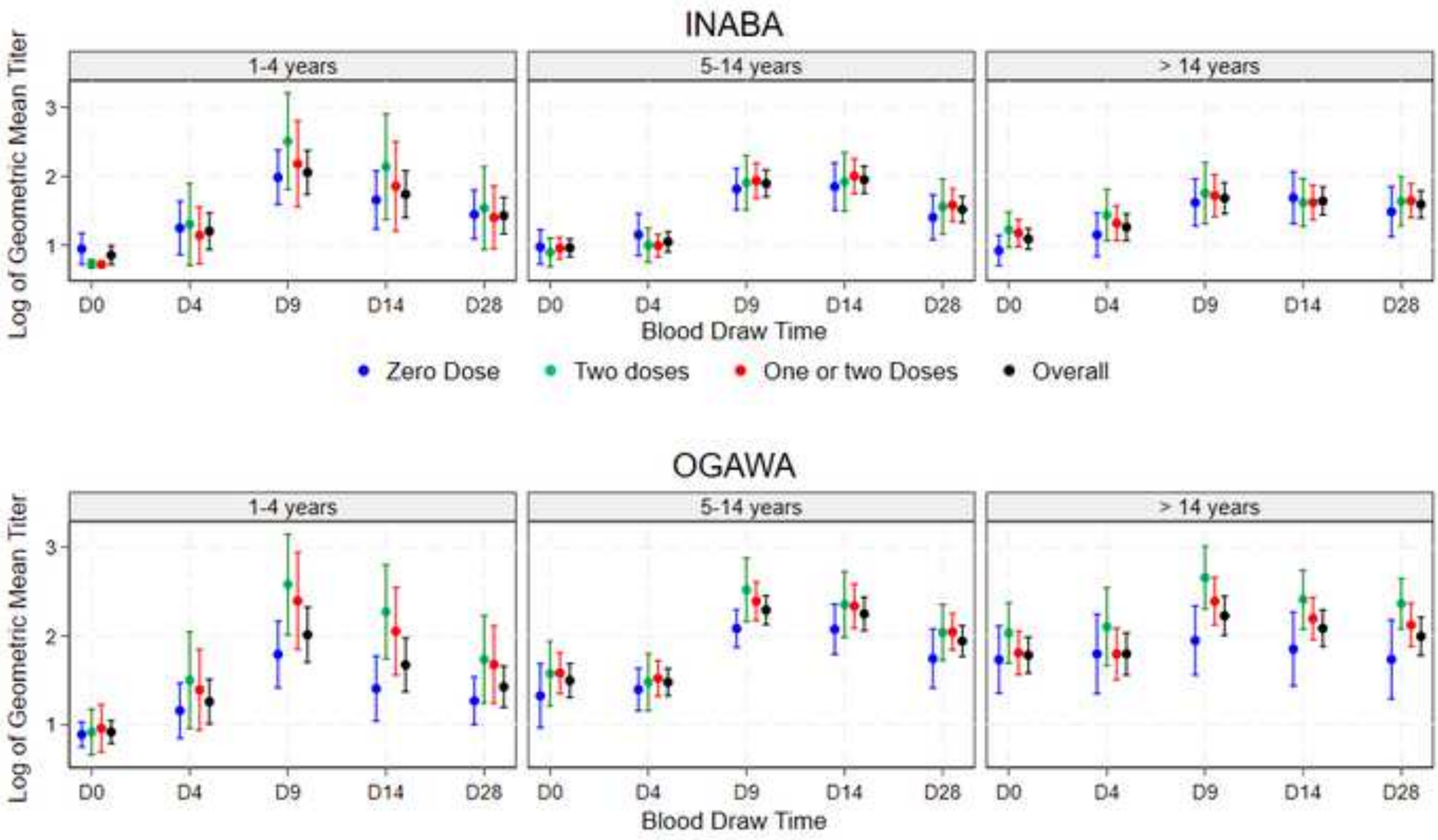

Figure 4-a: Vibriocidal GMT of the participants who received 1 or 2 OCV doses earlier in Arms A and B through the span of the study

Click here to access/download;Figure;Figure 5a.tif

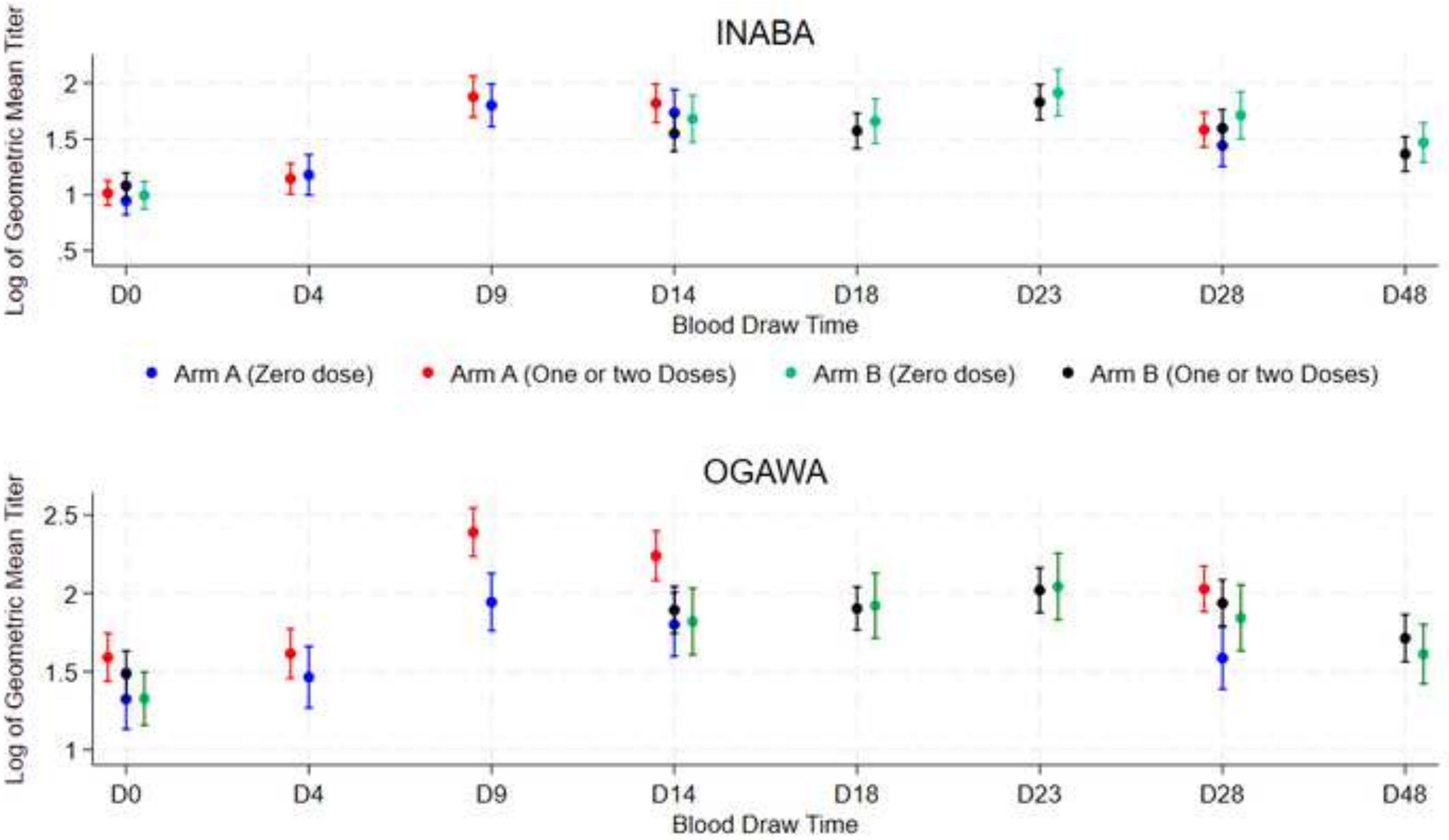

Click here to access/download
Supporting Information
Ethical Clearance French.pdf

Click here to access/download
Supporting Information
CONSORT_2025_checklist-MOBS.docx

Translated ethical clearance

Click here to access/download
Supporting Information
English ethical clearance MOBS.pdf

Trial protocol

Click here to access/download
Supporting Information
Protocole MOBS Dec 2020.pdf

Supporting information file 2: Annex tables

Click here to access/download
Supporting Information
Annex Tables.docx

Click here to access/download
Supporting Information
Base MOBS treated2_.dta

Title: Serological responses to killed oral cholera vaccine (OCV) when given 4 years after initial receipt of OCV in Cameroon: A randomized controlled trial.

Short title: Oral Cholera Vaccine booster dose serological response in Cameroon

Jerome Ateudjieu[1,2,3*], Ketina Hirma Tchio-Nighie[1,2], Etienne Guenou[1,2,6], Carrel Fokou[1], Collins Buh Nkum[1], Landry Beyala Bita'a[1], Winny Dora Ateudjieu-Kenfack[4,5], Sabine Nanfak[1], Amanda K Debes[7], David A Sack[7]

Affiliations

[1]M.A. SANTE, Yaounde, Cameroon

[2] Faculty of Medicine and Pharmaceutical Sciences, University of Dschang, Dschang, Cameroon.

[3]Clinical Research Unit, Division of Health Operations Research, Ministry of Public Health, Cameroon. Yaounde, Cameroon

[4] Department Microbiology, Faculty of Sciences University of Yaounde 1, Yaounde, Cameroon

[5]*Center of Research on Emerging and Reemerging Diseases, Yaounde, Cameroon*.

[6]National Public Health Laboratory, Ministry of Public Health, Yaounde, Cameroon.

[7]Johns Hopkins Bloomberg School of Public Health, Baltimore, Maryland. USA.

*Corresponding author

Jerome Ateudjieu: jateudj@masante-cam.org

Abstract

Background. Oral cholera vaccine (OCV) reduces the rates of cholera in populations at risk. Two doses are recommended for people living in hotspot areas as a preventative intervention. Since the vaccine is known to provide protection for 3 to 5 years, revaccination for these areas is warranted to continue to provide immunity. When revaccination is considered, there is a need for ~~of consistent~~ evidence guiding whether revaccination should use two doses or whether a single dose may be sufficient because this may induce a booster response. An OCV campaign was conducted in Mogode, Cameroon in 2017 which provided an opportunity to determine if this earlier OCV campaign would induce a booster response if revaccination was conducted four years later.

Methods. 350 people living in Mogode Cameroon, who received 0, 1 or 2 doses of OCV four years earlier ~~participated in a clinical trial~~ and, ~~-~~were ~~The groups were also~~ stratified into age groups, <5, 5-14, and >14 and were randomized into two arms. Arm ~~-~~A received a single dose and Arm B received two doses with the second dose given 14 days after the first. ~~-to determine age specific serological responses. .~~ . We aimed ~~planned~~ to determine if people who had received OCV four years earlier responded with a booster immune response (as suggested by a rapid rise in vibriocidal titer (by day 4 to 6)) compared to those who had not received OCV earlier. ~~-. The immune response was defined as indicated~~ as~~by a rise in vibriocidal titer within 4 -6 days of receiving OCV depending on whether the person had received zero, one, or two doses in 2017.~~ ~~-~~ T~~he participants were randomized into two groups, group A (single dose) and group B (two doses).~~ ~~-~~Serum, was collected at baseline for both groups. ~~-~~For Arm~~group~~ A, additional serum ~~Blood~~ samples were obtained ~~periodically~~ at day 4, 9, 14 and 28 following the first dose. ~~-~~For Arm~~group~~ B, additional serum samples were obtained on these same days, but after the second dose. Vibriocidal titers were determined for each of the serum samples. ~~-administrations of dose 1 in arm A and dose 2 in arm B OCV in both study groups to estimate immune response. The groups were also stratified into age groups, <5, 5-14, and >14 to determine age specific serological responses.~~

Formatted: Font: Bold

Results. Nine days after the OCV, vibriocidal GMT significantly increased in each of studythe armsgroups with a response rate of 61 to 70%; however, an early vibriocidal response on day 4-6 was rare (22%) and were the same in those who had received vaccine four years earlier and those who did not. -Ogawa, but not Inaba, vibriocidal responses were higher on day 9 for those who had been vaccinated earlier, but vibriocidal titers during subsequent timepoints were similar in all study armsgroups. Four to six days following the second dose (Armgroup B), there was no evidence of an early highery booster response compared to those of Arm A.-

Conclusion. An increase in the proportion of subjects showing an early booster vibriocidal serological response was not seen amongin groups of people who received OCV 4 years earlier compared to those who did not receive. The tendency was observed in each age group. Pour le serotype Ogawa, le peak vibriocidal serological response was higher in people vaccinated four year earlier compared to those not previously vaccinated. The tendence was observed in Arm A and Arm B. Further studies are needed to confirm these findings and guide the booster vaccination protocol using the OCV vaccine. This data suggests that when OCV campaigns are planned to revaccinate for populations living in hotspots that had received OCV earlier, the campaign should plan to provide two doses in the same way as OCV is given to groups that had not received OCV earlier.

Trial registration: Pan African Clinical Trials Registry under the reference PACTR202102660004195.

Formatted: English (United Kingdom)

Background

Cholera is currently one of the most alarming diseases with epidemic potential because of frequent outbreaks in vulnerable populations, rapid spread, high case-fatality rate, and negative impact on the performance of health systems. (1–3). Oral Cholera Vaccine (OCV) vaccination is one of the most effective responses to prevent cholera transmission, propagation, morbidity and mortality (4–6). While both killed whole cell and live attenuated vaccines have been licensed, only the killed oral whole cell vaccine is used through the global stockpile (7,8). This vaccine is recommended by the World Health Organization (WHO) in hotspots of cholera endemic countries, during humanitarian crises with

high a risk of cholera, and during cholera outbreaks (9–11). Since the duration of protection of OCV is estimated to be 3 to 5 years, the WHO recommends that revaccination may be needed after 3 years where there is continued risk of cholera (12). To date, the vaccine manufacturers have not issued any recommendations for booster vaccination for the killed whole cell cholera vaccine.

Two studies examined the response following a repeated (booster) dose. A study in Bangladesh found that children age <5 years had a higher serological response to a single dose of OCV as measured by geometric mean vibriocidal titers if they had received the vaccine three years earlier, but other age groups (age >5 years and adults) did not show this enhanced response (13). By contrast, a study from India~~n~~ did not find a difference in response to OCV when two doses of vaccine was given 5 years after the first vaccination (14). Neither of these studies examined the kinetics of the immune response, such as a rapid antibody response in the first week that may indicate a true booster response (15–17).

Because of an outbreak in the area, the Cameroon Ministry of Public Health and its partners carried out an OCV vaccination campaign in 2017 in Mogode Health District located in the Far North region of Cameroon. This was a two rounds immunization campaign using Shanchol that targeted people aged ≥1 year old. A coverage survey conducted between the two rounds and after the second round estimated that 80.1% of the population had received two doses and 4.3% had received one dose (18). Since the campaign, there have been no reports of cholera between 2017 and 2021 nor has there been any administration of cholera vaccinations by the Ministry of Health that may have confounded this analysis. This previous exposure of the populations of Mogode Health district to OCV vaccination provided an opportunity to explore the immune response of a booster OCV vaccination to answer the following questions. 1) When given OCV, do people who had received two OCV doses four years earlier develop a rapid (within 4 to 6 days) serum vibriocidal response more commonly than those who did not receive OCV four years earlier. 2) When given OCV, is the geometric mean titer (GMT) vibriocidal in each age group (1-4, 5-14, ≥15) superior in people who received OCV four years earlier compared to those who did not receive OCV four years earlier? 3) When given OCV, is the serum vibriocidal responses in children inferior to the response in older age groups? 4) Do the immune

responses after one or two doses OCV administration, differ in people exposed to one or two doses of OCV earlier?

It is anticipated that the findings from this study will help guide use of OCV when given to groups of people who were previously vaccinated. If persons previously vaccinated show evidence of a booster response, then it would seem that a single dose would provide sufficient immunity and that a second dose would not be needed. However, if a booster dose is not observed, this would suggest that such groups should be provided vaccine in the same way as groups that had not previously received vaccine.

Materials and Methods

Trial registration

The study protocol was registered in Pan African Clinical Trials Registry under the reference PACTR202102660004195(https://pactr.samrc.ac.za/TrialDisplay.aspx?TrialID=14625).

Study design

Individuals leaving in Mogode Health District aged four years and above and who had received zero, one or two doses of OCV during the 2017 OCV campaign were eligible to participate. Those fulfilling inclusion criteria were ~~randomly~~ stratified by age (4 years, 5-14 years, ≥15 years) and 2017 OCV exposure status (zero dose, one dose, two doses) and randomly~~were~~ assigned to receive one dose of OCV at day zero (Arm A) or two doses of OCV at day 0 and 14 (Arm B). Serum samples were collected day 4-6, 9-12, 14-16 and 28-30 following administrations of dose 1 in arm A and on day 4-6, 9-12, 14-16 and 28-30 after the second dose ~~dose 2 in~~ (arm B) ~~periodically~~ to determine and compare the overall serum vibriocidal responses and by age group according to 2017 pre-vaccination exposure status, and to compare the vibriocidal response per study arm.

Formatted: Highlight

Setting

The study was conducted in Mogode health district located in the Far North region of Cameroon. Mogode health district benefited from a two rounds OCV campaign in May and June 2017. The immunization coverage estimated from the end campaign survey was estimated for two, one and zero doses documented coverage at 80.1%, 4.3% and 3.8% respectively (18). Enrollment of participants for the present trial was conducted ~~between~~ from February ~~and~~ to June 2021. Between this campaign and the start of the study in 2021, no case~~s~~ of cholera were detected in Mogode health District or any of bordering health district by the surveillance system in place and no other campaign with OCV was conducted in the area during the interval. In the same period, no additional OCV vaccination activity was conducted in the Mogode Heath District or in any of bordering health District.

Figure 1: Location of the Mogode health district with its health areas.

Participants

All individuals aged 1 year and above during the 2017 OCV vaccination campaign living in the Mogode health district and who received either two, one or zero doses of OCV during the 2017 campaign were eligible. We excluded all individuals who reported to have received OCV vaccines after the 2017 OCV campaign before the study period, women who were pregnant, persons enrolled in another study, those with a history of diarrhea during the seven days prior to first dose of vaccine (defined as ≥3 loose stools in 24 hours) or history of chronic diarrhea (lasting for more than 2 weeks in the past 6 months), those currently using laxatives, antacids, or other agents to lower stomach acidity, those with chromic health conditions, or immunosuppression and persons not willing to provide informed consent.

Sample size

"To determine whether a rapid vibriocidal response following single dose OCV administration among those exposed to OCV four years earlier is superior to those exposed to zero doses four years earlier,

Formatted: Font: (Default) +Body (Calibri), 11 pt

Formatted: Line spacing: Double

we attempted to recruit 20 people for each age group (1-4, 5-14, ≥15 years old) in Arm A and the same number for Arm B. Among those who had received OCV four years earlier, we intended to evaluate each age group into those who had received zero, one dose or two doses earlier. Thus, total sample size was estimated to be 360 with 180 participants expected per study arm, including 60 per OCV vaccination status (0, 1 or 2 doses) distributed in 20 per age group. We hypothesized that an early vibriocidal response following single dose administration (Arm A) will occur among 25% of those in non-exposedcontrol group (those exposed to zero OCV dose four years earlier) and will be increased to 75% in the exposedintervention group (those exposed to 2 OCV doses four years earlier) (confidence=95%, power=90%), that early immune response in arm A will not differ to that of Arm B and that the dropout rate in each study arm age group will be 10% or less.Aee id “

Formatted: Font: (Default) +Body (Calibri), 11 pt, English (United States)

To determine whether a rapid vibriocidal response following single dose OCV administration among those exposed to 2 OCV doses four years earlier is superior to those exposed to zero doses four years earlier, we attempted to recruit 40 people for each age group (1-4, 5-14, ≥15 years old). We hypothesized that an early vibriocidal response will occur among 25% of those in control group (those exposed to zero OCV dose four years earlier) and will be increased to 70% in the intervention group (those exposed to two OCV doses four years earlier (confidence=95%, power=90%), and that the dropout rate in each age group will be 10%.

Study procedures

Community involvement. With the help of local health authorities, the traditional community leaders were invited to a meeting at which the rationale, objectives, implementation procedures and expectations of the project were presented prior to intervention implementation and discussed. The leaders understood the project, and each made a commitment to facilitate the project's activities in their respective communities. They contributed to the identification of community volunteers to be involved in project activities in their communities. These included persons able to speak local (Kapsiki) and an official language (English or French) living in one of targeted communities. These community

volunteers were trained to visit households, introduce the project to the head of household, and seek his permission to invite members of their household to visit the project site as potential participants. The community volunteers were also involved in reminding the people included in the study of their visit schedules.

Enrollment. Households in the Mogode health area in the Mogode health district were visited by community volunteers to introduce the project to household heads and obtain permission to brief household inhabitants and invite those eligible to the study site prepared for this purpose with the collaboration of the Mogode district hospital. Participants presenting themselves at the study site were informed about the study. Those who consented were screened for eligibility and selection criteria. Eligible participants meeting the inclusion~~ / exclusion~~ criteria were assigned to the two arms of the study by randomization. During the follow-up period, participants were reminded by telephone or through home visits of the dates and times of their follow-up visits~~ to the study sites~~. Enrollment of participants for the present trial was conducted from February to June 2021.

Determining participants age and prior vaccination status. For eligible participants, the age was collected on the identity card or birth certificate. In the absence of these documents, age was tracked using the documented ages of other children in the household or neighborhood, or using major events whose timing in the past was known. Vaccination status 4 years prior to OCV was determined using the vaccination record, or tracked using photos of the vaccine, the vaccine administration protocol and specific questions asked to participants or guardians (the period of the campaign, the presence of the participant in the community during the campaign, the description of the vaccine vial and the taste of the vaccine if possible).

Randomization. Randomization was stratified by age group and prior exposure status to OCV. Thus, in the stratum of each age group, participants who had received zero, one or two doses of OCV were randomly assigned in blocks of two to Arm A or Arm B. The team responsible for randomization was different from the team enrolling and randomly assigning participants to study arms. The

Formatted: Justified, Line spacing: Double

Formatted: Font: Not Bold

Formatted: Font: (Default) Calibri, 11 pt, Font color: Text 1

randomization procedure provided to generate the four possible combinations of blocks of two (AA, AB, BA, BB, each combination indicating the assignment of each pair of participants to the two study arms) and ranked in each stratum of of expected participants in random order and associated with numbers to generate unique code that indicted the study arm of each participant. The code was printed on a label, inserted into opaque envelopes arranged per stratum in order and numbered. The randomization was implemented by taking par stratum (age group and vaccination status) an envelope for an included patient following the order of numbers dictated by the randomization team, opening it, removing the label and assigning the number to the participant.

Formatted: Space After: 8 pt

Interventions

The administration of the vaccine was conducted at the study implementation site. Participants in arm A received oral administration of one dose of OCV (Shanchol, Lots 1: SCN006A19 and Lot 2: SCN034A20) on day zero. Participants in arm B received two doses of OCV (same batch); the first dose was on day 0 and the second dose was on day 14 to 16. Shanchol consists of a mixture of killed *V. cholerae* O1 and O139 in single-dose glass vial containing 1.5 mL of vaccine. The entire vial of OCV was administered orally to each participant irrespective of their age. The vaccine vial was shaken to ensure mixing and was inspected by the study nurse before administration. To monitor for safety, all participants were observed for 30 minutes after taking the vaccine and had to report any adverse events occurring within two weeks of receiving the vaccine to the community worker or during the next study planned visit. For ethical reasons, and to ensure that all study participants had an opportunity to receive the recommended two doses of vaccine, the participants in arm A were invited to take the second OCV dose after their follow-up period, which ended on day 28, but no additional serum samples were collected to assess the serological response after this second dose in arm A.

Outcome assessment

In arm A, participants were followed up for 28 days with five blood draws and in arm B, for 42 days with six blood draws. The timing of the follow-up vaccinations and blood draws along with the acceptable visit windows for each event are shown in figure 2~~1~~. The primary outcome was vibriocidal GMT and seroconversion rate measured four to six days following single dose OCV to assess and compare early immune responses between participants exposed to two doses OCV four years earlier to those exposed to zero doses four years earlier. Secondary outcomes included 1) Age group specific (1-4, 5-14, ≥15 years) and overall vibriocidal GMT and seroconversion rates measured at days 4-6, 9-12, 14-16 and 28-30 following OCV administration to compare vibriocidal responses between participants who received one or two OCV doses four years earlier and those who received zero doses four years earlier. 2) Vibriocidal GMT and seroconversion rates measured four days following a single OCV dose to compare early immune response between participants exposed to one or two doses of OCV four years earlier and those exposed to zero doses. 3) Vibriocidal GMT and seroconversion rate 14-16 days following exposure to the single dose (arms A and B) and days 18-20, 23-25, 28-30 and 42-44 for arm B.

Figure 2: Visit windows, vaccination and blood draw schedules

Laboratory procedures

The timing of the blood samples is shown in figure 2. Blood sample collection procedures depended on age group. For age group <5years, a fingerstick sample was obtained using a lancet and capillary tube to collect 350 µl of blood which was then diluted in 1400 µl normal saline which resulted in a 1:5 dilution of whole blood. For subjects ≥5 years 5 ml of venous blood was collected using a syringe. The blood samples were centrifuged and the serum was stored at -20°C until assayed.

For the vibriocidal assay, we used *V. cholerae* strains isolated from Cameroon (Inaba 600040 and Ogawa CPC-BACT-01) as previously described (19). The strains were incubated with serially diluted, heat-inactivated serum with exogenous guinea pig complement (Sigma Aldrich, catalogue # S234395) on a shaker (50 rev/min) at 37°C for 1 hour.[16] The starting dilution for the previously undiluted serum

was 1:10 while the previously diluted serum from young children was used without further dilution considering that the 1:5 dilution of whole blood to be equivalent to the 1:10 dilution of serum. Vibriocidal titers were defined as the reciprocal of the highest serum dilution which results in a 50% reduction in optical density (595 nm) compared with growth control without serum. Standard sera, comprising of high titer human serum and high titer rabbit serum were employed to normalize the outcomes and mitigate the effects of inter-experimental variation. Samples were tested in duplicates. Titers lower than the lowest detectable titer were assigned to have a titer equivalent to half of the lowest detectable titer. The threshold for inter- and intra-experimental variation was set at 2-fold. Seroconversion was defined as ≥ 4-fold rise in vibriocidal titers compared with baseline titer.

Data analysis

To identify confounders between OCV exposure status and between study arms we compared the distribution of participants' baseline characteristics between those exposed to zero OCV doses and those exposed to two doses four years earlier, and between those assigned to the two arms.

For the primary analysis, we compared the vibriocidal GMT and seroconversion rates on day 4-6 following the administration of a single dose of OCV between participants exposed to two OCV doses in 2017 and those exposed to zero doses using Student’s T test and Z test respectively. For the estimate of vibriocidal seroconversion rate, logistic regression model was used for the adjustment using individual and household characteristics as adjustment variables.

For the secondary analyses, we carried out the same analysis per age group between participants exposed to 1 or 2 OCV doses and those exposed to zero doses in 2017. These estimates were also conducted on day 9-12, 14-16, and 28-30 following the single OCV dose administration for participants of arm A. Diagrams showing estimated of vibriocidal GMTs with a 95% confidence interval on the planned blood draw days flowing single dose OCV administration (Arm A) and two doses OCV administration (arm B) was generated for overall, by age group and according to 2017 OCV vaccination statusThe estimate of vibriocidal GMT and seroconversion rate was also done at

Formatted: Space After: 8 pt

~~day 14-16 overall and per age group following single OCV dose administration for participants of both arm A and arm B and at days 18-20, 23-25, 28-30 and 42-44 for participants of arm B~~. The comparison of vibriocidal GMT was done using the Student T test and that of seroconversion rate using the Z test. Regarding the seroconversion rate.~~,~~ For the estimate of vibriocidal seroconversion rate, logistic regression model was used for the adjustment using individual and household characteristics as adjustment variables.

Formatted: Font: (Default) +Body (Calibri), 12 pt, Font color: Auto

~~the logistic regression model was used for the adjustment using individual and household characteristics as adjustment variables.~~ The comparison was made with one tailed 95% confidence interval. Analyses were conducted using Stata (Version 18).

Ethical Considerations

Permission from adequately informed community authorities was obtained before accessing eligible participants in households. All study participants were fully informed in an appropriate language about study justifications, objectives, procedures, implications, and expected results and were given the time to ask questions before deciding to consent or not to participate. Only those that provided their consent were included. For minor participants (<21 years), parental permission was obtained. For minors aged between 12 and 20, their assent was sought and obtained prior to their inclusion in the study. Their assent was accompanied by a parental consent. Measures were taken to ensure confidentiality of patient data during inclusion, administration of interventions, patient sampling, data processing and analysis. All participants were observed 30 minutes after vaccine administration and visited to detect, report and investigate any adverse events occurring after vaccine administration. Similarly, measures were taken regarding the risk of infection, trauma and pain resulting from biological sampling. The research protocol was approved the Cameroon National Ethics committee for Human Health Research (N°: 2020/12/1321/CE/CNERCH/SP).

Results

Enrollment

The consort flow diagram for the study is shown in Figure 3. Of the 430 persons who were received in the study site~~were approached~~, 3 did not consent to be screened and ~~, and~~ 77 did not meet inclusion~~/exclusion~~ criteria~~ and were excluded~~. 350 (81.4%) participants were enrolled in the study with 136, 101 and 113 with OCV vaccination status four years earlier of 0, 1 and 2 doses respectively. For those who were included, 174 and 176 were randomly assigned to Arm A and Arm B respectively.

Figure 3: Consort flow diagram

Baseline characteristics of participants regarding study arms.

Table 1 presents the comparison of participants' characteristics per study arm. No difference in the distribution of these characteristics was detected.

Table 1: Distribution and comparison of participants characteristics per study ~~cohort~~arm

**Characteristics**	**Arm A (N=174)**	**Arm B N(176)**
Sex, n(%)		
Male	72 (41.4)	69 (39.2)
Age group,n(%)		
1-4	37 (21.3)	37 (21.0)
5-14	69 (39.7)	69 (39.2)
≥15	68 (39.1)	70 (39.8)
Age median(IQR)		
	11 (23)	12 (25.5)
Floor material participants houses, n (%)		
Cement / Brick/Tiles	50 (28.7)	40 (22.7)
Using improved toilet facility, n (%)		
	19 (10.9)	15 (8.5)

Formatted: Centered

Formatted: Centered

Weight in kg, mean [CI]			
	34.4 [31.5, 37.4]	36 [33, 38.9]	
Body Mass Index, mean [CI]			
	16.8[16.2,17.4]	16.9[16.3,17.6]	
Baseline LVGMT Inaba, geometric mean [CI]			
	1[0.9,1.1]	1 [1,1.1]	
Baseline LVGMT Ogawa, geometric mean [CI]			
	1.5[1.4,1.6]	1.4[1.3,1.5]	
Vaccination status, n(%)			
0 dose	68 (39.1)	68 (38.6)	
1 dose	49 (28.7)	52 (29.6)	
2 doses	57 (32.8)	56 (31.8)	
**Characteristics**	**Cohort A (N=174)**	**Cohort B N(176)**	**p.value**
Sex, n(%)*			
Male	72 (41.4)	69 (39.2)	0.68
Age group,n(%)*			
1-4	37 (21.3)	37 (21)	0.99
5-14	69 (39.7)	69 (39.2)	
≥15	68 (39.1)	70 (39.8)	
Age median(mean)			
	11 (20.2)	12 (19.7)	0.79
Floor material, n (%) *			
Cement / Brick/Tiles	50 (28.7)	40 (22.7)	0.198
Using improved toilet facility, n (%) *			
	19 (10.9)	15 (8.5)	0.45

Formatted Table

Formatted Table

Formatted Table

Formatted Table

Formatted Table

~~Weight in kg, mean [CI]++~~			
	~~34.4 [31.5, 37.4]~~	~~36 [33, 38.9]~~	~~0.45~~
~~Baseline LVGMT Inaba, geometric mean [CI]+~~			
	~~2.3[2.1,2.5]~~	~~2.4 [2.2, 2.6]~~	~~0.18~~
~~Baseline LVGMT Ogawa, geometric mean [CI]+~~			
	~~3.4[3.1,3.7]~~	~~3.3[3, 3.5]~~	~~0.6~~
~~Vaccination status, n(%)*~~			
~~0 dose~~	~~68 (39.1)~~	~~68 (38.6)~~	~~0.96~~
~~1 dose~~	~~49 (28.7)~~	~~52 (29.6)~~	
~~2 doses~~	~~57 (32.8)~~	~~56 (31.8)~~	

~~+ P.value from Wilcoxon test, * P.value from chi-square,~~ LVGMT= log of vibriocidal Geometric mean titer, VSCR= vibriocidal seroconversion rate.

Comparison of characteristics of participants per OCV exposure status, four years earlier.

Table 2 shows a comparison of the distribution of the characteristics of participants exposed to two or at least one dose of OCV compared with those exposed to zero dose. The comparison reveals relative differences in distribution for age groups, housing materials~~, height and weight and baseline Ogawa vibriocidal GMTs~~ between those who did not receive OCV earlier and those who did.

Table 2: Baseline comparison of participants who received no OCV earlier and those who received one or two doses earlier.

Characteristics	Received zero doses earlier compared to those who received two doses earlier		Zero doses compared to those who received one or two doses earlier
	Zero dose (N=136)	Two doses(N=113)	One or two doses (N=214)

Formatted Table

Formatted Table

Formatted Table

Formatted Table

		-	-
Sex, n(%)			
Male	53 (39.0)	48 (42.5)	88 (41.1)
Age group n (%)			
1-4	44(32.4)	21 (18.6)	30 (14.0)
5-14	46 (33.8)	46 (40.7)	92 (43.0)
≥15	46 (33.8)	46 (40.7)	92 (43.0)
Age median(IQR)			
	9 (20.0)	12 (23.0)	13 (29.0)
Housing ~~Floor~~ material, n (%)			
Cement / Brick/Tiles	47 (34.6)	17 (15.0)	43 (20.1)
Using improved toilet facility n(%)			
Yes	18 (13.2)	9 (8.0)	16 (7.5)
Weight in kg, mean [CI]			
	31.9[28.6,35.3]	36[32.3, 39.7]	37.3 [34.6, 39.9]
Body Mass Index, mean [CI]			
	16.4[15.7,17.1]	17[16.2,17.9]	17.2[16.6,17.8]
Baseline ~~L~~VGMT Inaba , [CI]			
LVGMT Inaba	1[0.9,1.1]	1 [0.9,1.1]	1 [1,1.1]
VGMT Inaba	9.3[7.6,11.5]	9.8 [7.9,12.2]	11.2 [9.3,13.4]
Baseline LVGMT Ogawa , [CI]			
LVGMT Ogawa	1.3[1.2, 1.5]	1.5 [1.4, 1.7]	1.5[1.4, 1.6]
VGMT Ogawa	21.2[15.8, 28.3]	34.5 [24.6, 48.5]	34.5[27.0, 44.0]

Formatted: Left

Formatted: French (France)

Formatted: French (France)

Formatted: French (France)

Formatted: Left

~~++ P values are from Wilcoxon test, + Value are from t-test, * P values are from chi-square,~~ VGMT= vibriocidal Geometric mean titer

Comparison of the early (day 4-6) vibriocidal response in participants who received zero, one or two doses of OCV four years earlier.

The early immune responses (vibriocidal GMT and seroconversion rate) of participants (all age group, per age group and for serotypes Inaba and Ogawa) exposed to two or at least one dose of OCV versus those exposed to zero dose is presented in Table 3. Early rise~~s~~ in vibriocidal titers was~~ere~~ rare in participants exposed four years earlier to two OCV doses, one OCV dose and no difference was observed when compared to those who were not reported to be administered an OCV vaccine dose in 2017. This was consistent across age groups and serotypes (Ogawa and Inaba). ~~and no statistical differences in vibriocidal GMT or seroconversion rates were detected for the entire group or for each age group, or for each serotype (Inaba and Ogawa) between those who received zero doses earlier and those who received two doses earlier or those who received one or two doses earlier.~~

Table 3 A: Comparison of Vibriocidal GMT and Seroconversion rates 4-6 days after a single dose of OCV depending on receipt of OCV four years earlier.

Age group and outcome (VGMT and VSCR)	Early vibriocidal titer changes among those who received zero doses of OCV and those who received two doses earlier (Arm A)			Early vibriocidal titer changes among those who received zero doses of OCV and those who received one or two doses earlier (Arm A)	
	Zero dose	Two doses	P value (adjusted)	One or two doses	P value (adjusted)
**All age groupAll ages**	**N=59N=59**	**N=49N=49**		**N=92N=92**	
**LVGMT Inaba,[CI]+LVG MT Inaba ,[CI]+**	1.2 [1;1.4]2.7 [2.3;3.1]	1.2 [1;1.4]2.8 [2.3;3.3]	0.70.7	1.1 [1;1.3]2.6 [2.3;2.9]	0.80.8
**LVGMT Ogawa,[CI]+LVG MT Ogawa, [CI]+**	1.5 [1.3;1.7]3.4 [2.9;3.8]	1.7 [1.5;1.9]3.9 [3.4;4.5]	0.10.1	1.6 [1.5;1.8]3.7 [3.4;4.1]	0.20.2
**VSCR Inaba, %[CI]*VSCR Inaba, n (%) ***	23.7 [12.5;34.9]14 (23.7)	22.4 [10.3;34.6]11 (22.4)	0.9 (0.60)0.9 (0.6)	12 [5.2;18.7]11 (12.0)	0.1 (0.1)0.1 (0.2)
**VSCR Ogawa, %[CI]*VSCR Ogawa n (%) ***	15.3 [5.8;24.7]9 (15.3)	18.4 [7.1;29.6]9 (18.4)	0.70 (0.5)0.7 (0.4)	10.9 [4.4;17.4]10 (10.9)	0.4 (.60)0.4 (1)
		-			
**1-4 years1-4 years**	**N=17N=17**	**N=9N=9**		**N=13N=13**	

Formatted Table

Formatted: Font: Bold

Formatted: Font: Bold

Formatted: Font: Bold

Formatted: Font: Bold

Formatted: Font: Bold

Formatted Table

Formatted Table

Formatted: Font: Bold

Formatted: Font: Bold

Formatted: Font: Bold

Formatted: Font: Bold

Formatted: Font: Bold

**LVGMT Inaba,[CI]+**LVG MT Inaba [CI]+	1.2 [0.9;1.6]2.9 [2;3.8]	1.3 [0.70;1.9]3 [1.6;4.4]	0.60.6	1.1 [0.70;1.5]2.6 [1.7;3.6]	11
**LVGMT Ogawa,[CI]+**LVG MT Ogawa, [CI]+	1.2 [0.8;1.5]2.7 [2;3.4]	1.5 [1;2]3.5 [2.2;4.7]	0.20.2	1.4 [.9;1.8]3.2 [2.2;4.3]	0.40.4
**VSCR Inaba, % [CI]***VSCR Inaba, n (%) *	29.4 [5.3;53.6]5 (29.4)	33.3 [0-5.1;71.8]3 (33.3)	0.8(0.9)0.8(0.6)	23.1 [5.3;49.6]3 (23.1)	0.70 (0.70)0.7 (0.6)
**VSCR Ogawa, % [CI]***VSCR Ogawa n (%)*	29.4 [5.3;53.6]5 (29.4)	44.4 [3.9;85]4 (44.4)	0.4(.3)0.4(0.4)	30.8 [5.3;59.8]4 (30.8)	0.9 (0.60)0.9 (0.5)
**5-14 years5-14 years**	**N=22N=22**	**N=22N=22**		**N=42N=42**	
**LVGMT Inaba,[CI]+**LVG MT Inaba ,[CI]+	1.2 [.8;1.5]2.6 [1.9;3.4]	1 [0.8;1.2]2.3 [1.7;2.9]	0.30.3	1 [0.8;1.2]2.3 [1.9;2.7]	0.30.3
**LVGMT Ogawa,[CI]+**LVG MT Ogawa, [CI]+	1.4 [1.2;1.6]3.2 [2.7;3.8]	1.5 [1.2;1.8]3.4 [2.7;4.1]	0.90.9	1.5 [1.3;1.7]3.5 [3;4]	0.60.6

Formatted Table

Formatted Table

Formatted: Font: Bold

Formatted: Font: Bold

Formatted: Font: Bold

Formatted: Font: Bold

VSCR Inaba, % [CI]*VSCR Inaba, n (%)*	22.7 [3.7;41.7]5 (22.7)	18.2 [0.70;35.7]4 (18.2)	0.70(0.3)0.7(0.6)	9.5 [3.7;18.8]4 (9.5)	0.1 (0)0.1 (0.0)
VSCR Ogawa, % [CI]*VSCR Ogawa n (%)*	18.2 [0.70;35.7]4 (18.2)	13.6 [0-1.9;29.2]3 (13.6)	0.70(.2)0.7(0.3)	7.1 [.70;15.3]3 (7.1)	0.2 (0)0.2 (0.1)
-					
> 14 years> 14 years	N=20N=20	N=18N=18		N=37N=37	
LVGMT Inaba,[CI]+LVGMT Inaba ,[CI]+	1.2 [.8;1.5]2.6 [1.9;3.4]	1.4 [1.1;1.8]3.3 [2.4;4.2]	0.20.2	1.3 [1.1;1.6]3 [2.5;3.6]	0.40.4
LVGMT Ogawa,[CI]+LVGMT Ogawa, [CI]+	1.8 [1.4;2.2]4.1 [3.1;5.2]	2.1 [1.7;2.5]4.8 [3.8;5.9]	0.30.3	1.8 [1.5;2.1]4.1 [3.5;4.8]	0.90.9
VSCR Inaba, % [CI]*VSCR Inaba, n (%)*	20 [.8;39.2]4 (20)	22.2 [0.9;43.5]4 (22.2)	0.9(0.2)0.9(0.1)	10.8 [0.8;21.3]4 (10.8)	0.3 (1)0.3 (0.9)
VSCR Ogawa, % [CI]*VSCR Ogawa n (%)*	0 [0;0]0 (0)	11.1 [0-5;27.2]2 (11.1)	0.10.1	8.1 [0;17.3]3 (8.1)	0.2 0.2

+ P.value are from Wilcoxon test, * P.value are from chi-square, LVGMT= log of vibriocidal Geometric mean titer, VSCR= vibriocidal seroconversion rate. The logistic regression model has been used for the adjustment with seroconversion status as dependent variable, vaccination status in 2017 as main independent variable and other variables (Sex, Age group, floor material, listen information, using improved toilet facility, weight,

and height) as adjustment variables (age group was considered as adjustment variable for all agehas been included only at the global level).

Distribution and comparison of vibriocidal GMT and seroconversion rates 9-12 days after a single OCV dose depending on receipt of OCV four years earlier

Table 4 presents the comparison of vibriocidal GMT and seroconversion rate 9-12 days following single OCV dose depending on receipt of OCV earlier. -The Ogawa vibriocidal GMT at day 9-12 following a single OCV dose administration amongin all age group, and each age group significantly increased among people exposed four years earlier to zero, two doses and one or two OCV doses and was significantly higher in those exposed to one or two doses OCV. The similar tendency was observed for Inaba serotype but there was no difference comparing vibriocidal GMT in those exposed four years earlier to OCV to those not exposed. The increasing of vibriocidal seroconversion rate -for Ogawa and Inaba was also observed but no difference was detected between those exposed to OCV four years and those not exposed.

-was higher in participants who had received OCV four years earlier compared to those who did not receive OCV earlier, but the seroconversion rates were similar between the different groups. For the Inaba serotype, neither the vibriocidal seroconversion rate nor the GMTs were different for the groups. Figures 4-a and 4-b show that for all and each age groups, the vibriocidal GMT for samples collected 9-12, 14-14 and 28-30 days after receiving single OCV dose tended to be higher among those who received OCV earlier compared to those who did not receive OCV earlier.

Formatted: Justified

Table 4: Comparison of Vibriocidal GMT and Seroconversion rates 9-11 days after a single dose of OCV depending on receipt of OCV four years earlier.

Blood characteristics (outcomes) 9-11 days after the single dose administration	CohortArm A Number of OCV doses exposed four year earlier (zero dose and two doses)			CohortArm A Number of OCV doses exposed four year earlier (zero dose and one or two doses)	
	Zero dose	Two doses		One or two doses	

All age group	N=59	N=47	P-value (adjusted)	N=87	P-value (adjusted)
LVGMT Inaba,[CI]+LVGMT Inaba, [CI]+	1.8 [1.6;2]4.1 [3.7;4.6]	1.9 [1.7;2.2]4.5 [3.9;5.1]	0.50.5	1.9 [1.7;2.1]4.3 [3.9;4.7]	0.70.7
LVGMT Ogawa,[CI]+LVGMT Ogawa, [CI]+	1.9 [1.8;2.1]4.5 [4.1;4.9]	2.6 [2.4;2.8]5.9 [5.5;6.4]	00.0	2.4 [2.2;2.5]5.5 [5.1;5.9]	00.0
VSCR Inaba, %[CI]*VSCR Inaba, n (%) *	62.7 [50;75.4]37 (62.7)	68.1 [54.3;81.9]32 (68.1)	0.60 (0.4)0.6(0.5)	64.4 [54.1;74.6]56 (64.4)	0.8 (0.60)0.8(0.6)
VSCR Ogawa, %[CI]*VSCR Ogawa n (%) *	61 [48.2;73.8]36 (61.0)	70.2 [56.6;83.8]33 (70.2)	0.3 (0.2)0.3(0.2)	65.5 [55.3;75.7]57 (65.5)	0.60 (0.3)0.6(0.3)
**1-4 years1-4 years**	**N=19N=19**	**N=8N=8**		**N=11N=11**	
LVGMT Inaba,[CI]+LVGMT Inaba, [CI]+	2 [1.6;2.4]4.6 [3.7;5.5]	2.5 [1.8;3.2]5.8 [4.2;7.4]	0.20.2	2.2 [1.6;2.8]5 [3.6;6.4]	0.60.6
LVGMT Ogawa,[CI]+LVGMT Ogawa, [CI]+	1.8 [1.4;2.2]4.1 [3.3;5]	2.6 [2;3.1]5.9 [4.6;7.2]	00.0	2.4 [1.9;2.9]5.5 [4.3;6.8]	0.10.1
VSCR Inaba, % [CI]*VSCR Inaba, n (%) *	73.7 [51.9;95.5]14 (73.7)	100 [100;100]8 (100)	0.10.1	90.9 [51.9;111.2]10 (90.9)	0.3 (1)0.3(.)

Formatted Table

Formatted: Font: Bold

Formatted: Font: Bold

Formatted: Font: Bold

Formatted: Font: Bold

Formatted: Font: Bold

**VSCR Ogawa, % [CI]***VSCR Ogawa n (%) *	73.7 [51.9;95.5]14 (73.7)	100 [100;100]8 (100)	0.10.1	90.9 [51.9;111.2]10 (90.9)	0.3 (.60)0.3(1)
**5-14 years5-14 years**	**N=20N=20**	**N=21N=21**		**N=42N=42**	
**LVGMT Inaba,[CI]+**LVGMT Inaba, [CI]+	1.8 [1.5;2.1]4.2 [3.5;4.9]	1.9 [1.5;2.3]4.4 [3.5;5.3]	0.80.8	1.9 [1.7;2.2]4.4 [3.9;5]	0.60.6
**LVGMT Ogawa,[CI]+**LVGMT Ogawa, [CI]+	2.1 [1.9;2.3]4.8 [4.3;5.3]	2.5 [2.2;2.9]5.8 [5;6.6]	00.0	2.4 [2.2;2.6]5.5 [5;6]	00.0
**VSCR Inaba, % [CI]***VSCR Inaba, n (%) *	60 [36.5;83.5]12 (60)	71.4 [50.4;92.5]15 (71.4)	0.4(1)0.4(0.8)	73.8 [36.5;87.7]31 (73.8)	0.3 (0.70)0.3(0.4)
**VSCR Ogawa, % [CI]***VSCR Ogawa n (%) *	80 [60.8;99.2]16 (80)	71.4 [50.4;92.5]15 (71.4)	0.5(0.5)0.5(0.6)	66.7 [60.8;81.5]28 (66.7)	0.3 (0.3)0.3(0.4)
**> 14 years> 14 years**	**N=20N=20**	**N=18N=18**		**N=34N=34**	
**LVGMT Inaba,[CI]+**LVGMT Inaba, [CI]+	1.6 [1.3;2]3.7 [2.9;4.5]	1.8 [1.3;2.2]4 [3;5.1]	0.70.7	1.7 [1.4;2]4 [3.3;4.7]	0.70.7
**LVGMT Ogawa,[CI]+**LVGMT Ogawa, [CI]+	1.9 [1.6;2.3]4.5 [3.6;5.4]	2.7 [2.3;3]6.1 [5.3;6.9]	00.0	2.4 [2.1;2.7]5.5 [4.9;6.1]	0.10.1

Formatted: Font: Bold
Formatted: Font: Bold
Formatted: Font: Bold
Formatted: Font: Bold
Formatted: Font: Bold

Formatted: Font: Bold
Formatted: Font: Bold
Formatted: Font: Bold
Formatted: Font: Bold
Formatted: Font: Bold

**VSCR Inaba, % [CI]***VSCR Inaba, n (%) *	55 [31.1;78.9]11 (55)	50 [24.4;75.6]9 (50)	0.8(0.70)0.8(1)	44.1 [31.1;61.7]15 (44.1)	0.4 (0.5)0.4(0.7)
**VSCR Ogawa, % [CI]***VSCR Ogawa n (%) *	30 [8;52]6 (30)	55.6 [30.1;81]10 (55.6)	0.1(0.3)0.1(0.3)	55.9 [8;73.5]19 (55.9)	0.1 (0.1)0.1(0.1)

+ P.value from Wilcoxon test, * P.value from chi-square, LVGMT= Log of vibriocidal Geometric mean titer, VSCR= vibriocidal seroconversion rate The logistic regression model has been used for the adjustment with seroconversion status as dependent variable , vaccination status in 2017 as main independent variable and other variables (Sex, Age group, floor material, listen information, using improved toilet facility ,weight, and height) as adjustment variables (age group has been included only at the global level).

Trends of vibriocidal GMT following single dose administration of OCV

Formatted: Font: Bold

Figures 4-a and 4-b show that for all and each age groups, the vibriocidal GMT for Inaba serotype at days 9-12, 14-14 and 28-30 days following single OCV dose administration was not higher in participants exposed to two OCV doses, one or two OVC doses four years earlier than those who did received OCV at that period. The vibriocidal GMT for Ogawa serotype at days 9-12, 14-14 and 28-30 days following single OCV dose administration was higher in participants exposed to two OCV doses, one or two OCV doses four years earlier than those who did received OCV at that period. This tendency was observed for all and each age group.

Figure 4-a: Vibriocidal Geometric Mean Titer following single OCV of the three participating OCV status four year earlier through the span of the study.

Figure 4-b: Age stratified vibriocidal Geometric Mean Titer following single dose administration of the three participants OCV status four year earlier through the span of the study.

Trends of vibriocidal GMT and seroconversion in study arms A and B

The GMT and the two study arms are shown in Figure 5. For both Ogawa and Inaba serotypes, for all ages and for each age group and whether the subject had received OCV earlier, the administration of the second dose of OCV (arm B) did not lead to an increase in GMT vibriocidal values. No difference was detected between vibriocidal GMT titre of arm A compared to arm B in participants exposed to one or two OCV doses four years earlier; as was the case in those who had not been vaccinated four years earlier.

Formatted: Font: Not Bold

Formatted: English (United Kingdom)

Figure 5-a: Vibriocidal GMT of the participants who received 1 or 2 OCV doses earlier in Arms A and B through the span of the study.

Figure 5-b: Age stratified Vibriocidal GMT of the participants who received 1 or 2 OCV doses earlier in Arms A and B through the span of the study.

Comparison of Vibriocidal GMT and Seroconversion rates 28-30 days between arm A and arm B. ~~after the first dose of OCV~~

Table 5 presents the comparison between arm A and arm B of Vibriocidal GMT and Seroconversion rates 28-30 days from day 0~~after the first dose of OCV arm A and arm B~~. There was no difference between arms A and B for all and each age group or serotype.

Table 5: Comparison of Vibriocidal GMT and Seroconversion rates 28-30 days after the first dose of OCV arm A and arm B.

**Participants**	**~~Cohort~~Arm A and ~~cohort~~arm B immune response 28-30 days after the first dose administration**

	CohortArm A	CohortArm B	P-value (adjusted)
All age groupAll age group	N=147N=147	N=150N=150	-
LVGMT Inaba,[CI]+LVGMT Inaba, [CI]+	1.5 [1.4;1.6]3.5 [3.2;3.8]	1.6 [1.5;1.8]3.8 [3.5;4.1]	0.20.2
LVGMT Ogawa,[CI]+LVGMT Ogawa, [CI]+	1.9 [1.7;2]4.3 [4;4.5]	1.9 [1.8;2]4.4 [4.1;4.7]	0.60.6
VSCR Inaba, %[CI]*VSCR Inaba, n (%) *	49.7 [41.5;57.8]73 (49.7)	50.3 [42.2;58.5]75 (50.3)	0.9 (0.8)0.9 (0.8)
VSCR Ogawa, %[CI]*VSCR Ogawa n (%) *	33.3 [25.6;41]49 (33.3)	43.3 [35.3;51.4]65 (43.3)	0.1 (0.1)0.1 (0.1)
-	-	-	-
1-4 years1-4 years	N=31N=31	N=33N=33	-
LVGMT Inaba,[CI]+LVGMT Inaba, [CI]+	1.4 [1.2;1.7]3.3 [2.7;3.9]	1.3 [1.1;1.5]3 [2.4;3.6]	0.50.5
LVGMT Ogawa,[CI]+LVGMT Ogawa, [CI]+	1.4 [1.2;1.7]3.3 [2.8;3.8]	1.4 [1.2;1.6]3.2 [2.7;3.7]	11

Formatted Table

**VSCR Inaba, % [CI]***~~VSCR Inaba, n (%) *~~	45.2 [26.6;63.7]~~14 (45.2)~~	31.3 [14.3;48.2]~~10 (31.3)~~	0.3 (0.2)~~0.3 (0.2)~~
**VSCR Ogawa, % [CI]***~~VSCR Ogawa n (%) *~~	38.7 [20.5;56.9]~~12 (38.7)~~	45.5 [27.5;63.4]~~15 (45.5)~~	0.60 (0.60)~~0.6 (0.6)~~
-	-	-	-
**5-14 years**~~5-14 years~~	N=61~~**N=61**~~	N=63~~**N=63**~~	-
**LVGMT Inaba,[CI]+**~~LVGMT Inaba, [CI]+~~	1.5 [1.3;1.7]~~3.5 [3.1;3.9]~~	1.9 [1.7;2.1]~~4.3 [3.8;4.8]~~	0~~0.0~~
**LVGMT Ogawa,[CI]+**~~LVGMT Ogawa, [CI]+~~	1.9 [1.8;2.1]~~4.5 [4.1;4.9]~~	2.1 [2;2.3]~~4.9 [4.5;5.3]~~	0.1~~0.1~~
**VSCR Inaba, % [CI]***~~VSCR Inaba, n (%) *~~	50.8 [37.9;63.7]~~31 (50.8)~~	63.5 [51.3;75.7]~~40 (63.5)~~	0.2 (0.1)~~ 0.2 (0.1)~~
**VSCR Ogawa, % [CI]***~~VSCR Ogawa n (%) *~~	41 [28.3;53.7]~~25 (41)~~	50.8 [38.1;63.5]~~32 (50.8)~~	0.3 (0.2)~~0.3 (0.2)~~
-	-	-	-
**> 14 years**~~> 14 years~~	N=55~~**N=55**~~	N=54~~**N=54**~~	-
**LVGMT Inaba,[CI]+**~~LVGMT Inaba, [CI]+~~	1.6 [1.4;1.8]~~3.7 [3.2;4.1]~~	1.6 [1.4;1.8]~~3.7 [3.2;4.1]~~	1~~1~~

**LVGMT Ogawa,[CI]+**~~LVGMT Ogawa, [CI]+~~	2 [1.8;2.2]~~4.6 [4.1;5.1]~~	1.9 [1.7;2.1]~~4.4 [3.9;4.9]~~	0.7~~0.7~~
**VSCR Inaba, % [CI]***~~VSCR Inaba, n (%) *~~	50.9 [37.3;64.5]~~28 (50.9)~~	46.3 [32.6;60]~~25 (46.3)~~	0.60 (0.60)~~0.6 (0.6)~~
**VSCR Ogawa, % [CI]***~~VSCR Ogawa n (%) *~~	21.8 [10.6;33.1]~~12 (21.8)~~	33.3 [20.3;46.3]~~18 (33.3)~~	0.2 (0.1)~~0.2 (0.1)~~

+ P-value from Wilcoxon test, * P-value from chi-square, LVGMT= Log of vibriocidal Geometric mean titer, VSCR= vibriocidal seroconversion rate. The logistic regression model has been used for the adjustment with seroconversion status as dependent variable, vaccination status in 2017 as main independent variable and other variables (Sex, Age group, floor material~~, listen information~~, using improved toilet facility, weight, and height) as adjustment variables (age group has been included only at the global level).

Detected adverse events following OCV administration.

Formatted: Font: Bold

Five adverse events were detected including 2 cases of nausea in the 30 minutes following immunization and 03 cases of diarrhea in the fourteen days following OCV administration. None of these cases was serious.

Discussion

The present study was intended to identify a rapid response to a dose of OCV if the subject had received the OCV vaccine earlier indicating that these people had responded with a booster response. However, we did not observe rapid responses in those who had received OCV earlier. In fact, an early response was seen rarely in any of the groups and there was not a significant difference in the proportion with a rapid response between those who had received OCV earlier and those who did not.

This finding was consistent for each of age group (1-4, 5-14, ≥15 years old) and for both Ogawa and Inaba vibriocidal assays.

We did observe a higher peak Ogawa vibriocidal GMT after the initial dose OCV but not a higher Inaba GMT in those who had received OCV earlier compared to those who had not received OCV earlier. This was observed both for the entire age group and for age specific groups.

In Arm B, there were no rapid responses on day 18 (4 to 6 days after dose 2) to suggest a booster response to this second dose. The GMT at day 14, 28-days following administration of one dose of OCV in arm A and two doses 14 days apart in arm B did not differ for overall participants, for each age group, serotype Ogawa and Inaba and by prior exposure to OCV vaccine status.

The administration of two doses Oral Cholera Vaccine (OCV) is known to protect about 65% and a lower percentage of children aged 1-4 years for five years following the vaccination (12). The present study was planned with the hypothesis that the magnitude and speed of the immune response following OCV vaccination would correlate with immune memory and would therefore be higher and faster in previously vaccinated individuals than in those not previously vaccinated. The vibriocidal GMT and seroconversion rate for the Ogawa and Inaba serotypes was not higher for overall participants nor for each of the three age groups among those exposed four years earlier to one or two doses compared to those exposed to zero OCV dose four years earlier. These results are consistent with those of a study conducted in Bangladesh measuring immune response in all age groups on day 3 following administration of a single dose of Shanchol vaccine (13). The present study is the first study to examine these responses in these age groups, ≤5 years, 5-14 and ≥ 15 years) in people who had received two doses of OCV vaccine 4 years earlier. It is unlikely that not detecting differences of GMT and response rates between participants exposed to one or two doses of OCV and those exposed to zero doses be explained by differences in the distribution of some characteristics between exposure status at baseline, since these differences were taken into account in the adjustment of the estimation of the comparison tests. Similarly, it is unlikely that repeated, low-grade or undetected natural exposure to Vibrio cholerae in the study setting have reduced the differences in vibriocidal antibody

Formatted: Font: (Default) Calibri, 11 pt, Font color: Text 1

responses between vaccinated and unvaccinated participants. Following the cholera vaccination campaign organised in 2017, our team conducted a year-long epidemiological surveillance of cholera, screening suspected cases using rapid diagnostic tests for cholera. During this surveillance period, no positive case was detected.

A number of assumptions that may explain the lack of difference in early immune response according to the status of OCV vaccination status deserve to be reviewed. The relatively high frequency of malnutrition in the Far North Cameroun region, as documented by recent demographic health surveys, could explain the low immune response in people who have been previously vaccinated, but in this case, a difference in immune response in adults would have been observed (20). The vaccination status of most of the participants was not based on vaccination cards as these cards were no longer available but determined using process inspired from a previous study that was documented reliable in assessing immunization status in people vaccinated with no evidence (21). Despite the fact that the association between vibriocidal antibody titers and cholera immunity is not absolute, the present result suggests that early immune response resulting from single OCV administration is not sufficient to ensure cholera protection in all age groups (22). This trend should be confirmed exploring in the same context the use of other cholera vaccine immunity markers which appear in studies conducted in other contexts to show a higher booster immune response in populations previously exposed to vaccination (13).

The immune response following administration of a dose of OCV at 9-11 days after administration was higher for serotype Ogawa in each of the age groups of individuals previously exposed to cholera vaccination and was not higher for Inaba. The difference in kinetics of Inaba and Ogawa serotypes may be related to the earlier exposure the Ogawa serotype that was identified during the 2017 outbreak.(23–25). The immune response curve following the first dose of OCV vaccine shows an increasing vibriocidal concentration, peaking at 9-11 days after a single dose OCV administration and then gradually decreasing. The curve following the second dose peaks days 9-11 and days 23-25 but on days 28-30, the immune response among those previously exposed to OCV was not different

among those exposed to 1 dose OCV compared to those exposed to two doses. To the best of our knowledge, no study describing the booster immune response curve following the second booster dose has been published, nor has a study reported the booster immune response following the first and second booster doses of OCV in the same population. The data collected in our study do not allow to explain the absence of difference in booster immune response at 28-day following one and two doses of OCV vaccination. This lack of difference may be explained by a local intestinal immune response that is probably triggered by the OCV first dose that partially neutralizes the second dose to reduce its bioavailability and the resulting immune response (26). The consistency of the observed findings needs to be explored in different setting and over other biomarkers to guide the planning of revaccination populations in need.

In summary, we had hoped to observe a rapid vibriocidal response to participants who had been vaccinated four years earlier. We also intended to determine if the magnitude of the vibriocidal response was of higher in those vaccinated earlier. However, we did not observe a rapid vibriocidal response in any of the groups and there was no difference in this response depending on earlier vaccination. Similarly, except for a higher Ogawa in those vaccinated earlier, the vibriocidal titers were similar in the different groups. The study does have implications for determining the number of doses of vaccine to be delivered when revaccinating populations that were vaccinated earlier. Since the previously vaccinated people responded in the same way as those not previously vaccinated, we feel this data suggests that that re-vaccination of previously vaccinated groups should follow the same recommendations as vaccination of groups not previously vaccinated; that is, that two doses is appropriate whenever this is feasible.

The study presented some limitations. The study took place at the start of the COVID-19 pandemic in Cameroon, which led to delays in the supply of vaccines, reducing the enrolment window for eligible participants in the 1-5 age group in 2017. This resulted in the sample size for this age group not being reached. Also, data on the nutritional status of participants that could have contributed in guiding the understanding of the immune response in the study arms were not collected.

Acknowledgements. We acknowledge and thank the participants in Mogode who participated in this research study.

Potential conflicts of interest. All authors: No reported conflicts of interest.

Data availability: Data presented and analyzed for the present manuscript are available as "Supplementary information file 1"

Funding:

"This work was supported by a grant from the Wellcome Trust to JA (grant number: 215663/Z/19/Z) and a grant from the National Institute of Allergy and Infectious Disease to DAS (5R01AI123422). The Wellcome Trust provided suggestions as to the study design, but neither funder had any role in data collection and analysis, decision to publish, or preparation of the manuscript."

~~This work was supported by the Department for International Development and Wellcome Trust: grant number: 215663/Z/19/Z (which supported study design, application for ethical evaluation; the purchase of vaccines, consumables, field logistics; supervision and transport of samples; data collection and analysis, decision to publish, preparation of the manuscript) and the National Institute of Allergy and Infectious Disease (which supported supported part of laboratory equipment, the training of lab teams supervision of study planning and results dissemination)~~

~~(5R01AI123422).~~

Formatted: Default Paragraph Font, Font: (Default) Calibri, 11 pt, Font color: Text 1

Formatted: Justified, Line spacing: Double

Formatted: Font: (Default) Calibri, 11 pt, Font color: Text 1

References

1. Kanungo S, Azman AS, Ramamurthy T, Deen J, Dutta S. Cholera. The Lancet. 2022 Apr 9;399(10333):1429–40.

2. World Health Organization. Weekly Epidemiological Record, 2023, vol. 98, 38 [full issue]. Wkly Epidemiol Rec Relevé Épidémiologique Hebd. 2023 Sep 22;98(38):431–52.

3. World Health Organization. Weekly Epidemiological Record, 2024, vol. 99, 36 [full issue]. Wkly Epidemiol Rec Relevé Épidémiologique Hebd. 2024 Sep 6;99(36):479–96.

4. Pezzoli L. Global oral cholera vaccine use, 2013–2018. Vaccine. 2020 Feb 29;38:A132–40.

5. Shaikh H, Lynch J, Kim J, Excler JL. Current and future cholera vaccines. Vaccine. 2020 Feb 29;38:A118–26.

6. Wierzba TF. Oral cholera vaccines and their impact on the global burden of disease. Hum Vaccines Immunother. 2019;15(6):1294–301.

7. Song KR, Lim JK, Park SE, Saluja T, Cho SI, Wartel TA, et al. Oral Cholera Vaccine Efficacy and Effectiveness. Vaccines. 2021 Dec 15;9(12):1482.

8. Msyamboza KP, M'bang'ombe M, Hausi H, Chijuwa A, Nkukumila V, Kubwalo1 HW, et al. Feasibility and acceptability of oral cholera vaccine mass vaccination campaign in response to an outbreak and floods in Malawi. Pan Afr Med J [Internet]. 2016 Apr 20 [cited 2024 Oct 21];23(203). Available from: https://www.panafrican-med-journal.com//content/article/23/203/full

9. P S, Ck O, K R. A Quantitative Systematic Review and Meta-Analysis of the Effectiveness of Oral Cholera Vaccine as a Reactive Measure in Cholera Outbreaks. Prehospital Disaster Med [Internet]. 2018 Feb [cited 2024 Oct 21];33(1). Available from: https://pubmed.ncbi.nlm.nih.gov/29317005/

10. Wierzba TF. Oral cholera vaccines and their impact on the global burden of disease. Hum Vaccines Immunother. 2018 Oct 12;15(6):1294–301.

11. Amisu BO, Okesanya OJ, Adigun OA, Manirambona E, Ukoaka BM, Lawal OA, et al. Cholera resurgence in Africa: assessing progress, challenges, and public health response towards the 2030 global elimination target. Infez Med. 32(2):148–56.

12. World Health Organization. Cholera vaccine: WHO position paper, August 2017 – Recommendations. Vaccine. 2018 Jun 7;36(24):3418–20.

13. Chowdhury F, Bhuiyan TR, Akter A, Bhuiyan MS, Khan AI, Tauheed I, et al. Augmented immune responses to a booster dose of oral cholera vaccine in Bangladeshi children less than 5 years of age: Revaccination after an interval of over three years of primary vaccination with a single dose of vaccine. Vaccine. 2020 Feb 11;38(7):1753–61.

14. Kanungo S, Desai SN, Saha J, Nandy RK, Sinha A, Kim DR, et al. An Open Label Non-inferiority Trial Assessing Vibriocidal Response of a Killed Bivalent Oral Cholera Vaccine Regimen following a Five Year Interval in Kolkata, India. PLoS Negl Trop Dis. 2015 May 29;9(5):e0003809.

15. Rahman A, Rashu R, Bhuiyan TR, Chowdhury F, Khan AI, Islam K, et al. Antibody-Secreting Cell Responses after Vibrio cholerae O1 Infection and Oral Cholera Vaccination in Adults in Bangladesh. Clin Vaccine Immunol. 2013 Oct;20(10):1592–8.

16. Ritter AS, Chowdhury F, Franke MF, Becker RL, Bhuiyan TR, Khan AI, et al. Vibriocidal Titer and Protection From Cholera in Children. Open Forum Infect Dis. 2019 Apr 1;6(4):ofz057.

17. Bi Q, Ferreras E, Pezzoli L, Legros D, Ivers LC, Date K, et al. Protection against cholera from killed whole-cell oral cholera vaccines: a systematic review and meta-analysis. Lancet Infect Dis. 2017 Oct 1;17(10):1080–8.

18. Ateudjieu J, Yakum MN, Goura AP, Tembei Ayok M, Guenou E, Kangmo Sielinou CB, et al. An innovative approach in monitoring oral cholera vaccination campaign: integration of a between-round survey. BMC Public Health. 2022 Feb 5;22(1):238.

19. Ateudjieu J, Sack DA, Nafack SS, Xiao S, Tchio-Nighie KH, Tchokomeni H, et al. An Age-stratified, Randomized Immunogenicity Trial of Killed Oral Cholera Vaccine with Delayed Second Dose in Cameroon. Am J Trop Med Hyg. 2022 Nov 14;107(5):974–83.

20. Institut National de la Statistique (INS), ICF. Enquête Démographique et de Santé du Cameroun 2018 [Internet]. 2020. Available from: https://dhsprogram.com/pubs/pdf/FR360/FR360.pdf

21. Ateudjieu J, Tchio-Nighie KH, Yakum MN, Goura AP, Amada L, Sonkoua I, et al. Innovative approach in assessing the children's immunization status when it cannot be documented. J Public Health Afr. 2023 Sep 30;14(9):7.

22. Iyer AS, Harris JB. Correlates of Protection for Cholera. J Infect Dis. 2021 Dec 15;224(Supplement_7):S732–7.

23. Debes AK, Ateudjieu J, Guenou E, Lopez AL, Bugayong MP, Retiban PJ, et al. Evaluation in Cameroon of a Novel, Simplified Methodology to Assist Molecular Microbiological Analysis of V. cholerae in Resource-Limited Settings. PLoS Negl Trop Dis. 2016 Jan 6;10(1):e0004307.

24. Weil AA, Becker RL, Harris JB. Vibrio cholerae at the Intersection of Immunity and the Microbiome. mSphere. 2019 Nov 27;4(6):e00597.

25. Honda K, Takeda K. Regulatory mechanisms of immune responses to intestinal bacteria. Mucosal Immunol. 2009 May 1;2(3):187–96.

26. Liu Y, Zhou J, Yang Y, Chen X, Chen L, Wu Y. Intestinal Microbiota and Its Effect on Vaccine-Induced Immune Amplification and Tolerance. Vaccines. 2024 Aug;12(8):868.

Response to Reviewers

PGPH-D-25-01336

Serological responses to killed oral cholera vaccine (OCV) when given 4 years after initial receipt of OCV in Cameroon

	Comments	Responses
	**Journal Requirement**	
1	Please provide additional details regarding participant consent. In the ethics statement in the Methods and online submission information, please ensure that you have specified (1) whether consent was informed and (2) what type you obtained (for instance, written or verbal, and if verbal, how it was documented and witnessed). If your study included minors, state whether you obtained consent from parents or guardians. If the need for consent was waived by the ethics committee, please include this information.<br><br>If you are reporting a retrospective study of medical records or archived samples, please ensure that you have discussed whether all data were fully anonymized before you accessed them and/or whether the IRB or ethics committee waived the requirement for informed consent. If patients provided informed written consent to have data from their medical records used in research, please include this information.	The methods section of the manuscript includes an ethical consideration section that gives all details about the consent process during the study. Informed and written consent was obtained for all included participants. For participants aged below 12, parental permission was obtained. For participants aged 12-20 years, the assent was obtained from the participant accompanied by a parental/guardian permission. For individuals aged 20 years and above, written consent was obtained from the participant. This is documented from line 258 to 265.<br><br>We are not reporting retrospective study of medical records or archived samples.
2	We are unable to open your Supporting Information file "Base MOBS treated.dta". Please	We uploaded a new version of the database. The previous version of the database was made using Stata18, hence the difficulty to open with other versions of the software. To avoid

	kindly revise as necessary and re-upload.	this, we saved the data in the way that the old version of Stata will be able to open.
3	Please provide separate figure files in .tif or .eps format.	New versions of the figures have been uploaded in the indicated formats.
4	We have amended your Competing Interest statement to comply with journal style. We kindly ask that you double check the statement and let us know if anything is incorrect.	We approve the amended version.
5	Your current Financial Disclosure states, "This work was supported by the Department for International Development and Wellcome Trust: grant number: 215663/Z/19/Z and National Institute of Allergy and Infectious Disease (5R01AI123422)". However, your funding information on the submission form indicates that you received funding from "Wellcome Trust" with grant number "215663/Z/19/Z". Please indicate by return email the full and correct funding information for your study and confirm the order in which funding contributions should appear. Please be sure to indicate whether the funders played any role in the study design, data collection and analysis, decision to publish, or preparation of the manuscript.	This work was supported by a grant from the Wellcome Trust to JA (grant number: 215663/Z/19/Z) and a grant from the National Institute of Allergy and Infectious Disease to DAS (5R01AI123422). The Wellcome Trust provided suggestions as to the study design, but neither funder had any role in data collection and analysis, decision to publish, or preparation of the manuscript.<br>This was adjusted in the manuscript from line 444-447.
6	Some material included in your submission may be copyrighted. According to PLOS's copyright policy, authors who use figures or other material (e.g., graphics, clipart, maps) from another author or copyright holder must demonstrate or obtain permission to publish this material under the Creative Commons Attribution 4.0 International (CC BY 4.0) License used by PLOS journals. Please closely review the details of	The map of figure 1 was created using the software QGIS 2.18. The base map shapefile and layers were obtained from the Health Information Unit of the Cameroon Ministry of Public Health which is provided upon request. The figure can be deleted in case it still represents an issue.

	PLOS's copyright requirements here: PLOS Licenses and Copyright. If you need to request permissions from a copyright holder, you may use PLOS's Copyright Content Permission form.<br><br>Please respond directly to this email or email the journal office and provide any known details concerning your material's license terms and permissions required for reuse, even if you have not yet obtained copyright permissions or are unsure of your material's copyright compatibility.<br><br>Potential Copyright Issues: Figure 1: please (a) provide a direct link to the base layer of the map (i.e., the country or region border shape) and ensure this is also included in the figure legend; and (b) provide a link to the terms of use / license information for the base layer image or shapefile. We cannot publish proprietary or copyrighted maps (e.g. Google Maps, Mapquest) and the terms of use for your map base layer must be compatible with our CC-BY 4.0 license.<br><br>Note: if you created the map in a software program like R or ArcGIS, please locate and indicate the source of the basemap shapefile onto which data has been plotted.	
7	If the reviewer comments include a recommendation to cite specific previously published works, please review and evaluate these publications to determine whether they are relevant and should be cited. There is no requirement to cite these works unless the editor has indicated otherwise.	There was no recommendation requesting to cite specific previously published work.
	**Additional comments**	
1	Line 98: The phrase "randomly stratified by age" appears ambiguous. Stratification and randomization are typically distinct steps in study design. Could the authors clarify whether participants were stratified by age	Participants were organized in stratus of age groups (1-4 year, 5-14, ≥15 years old) and randomization was done independently in each of the age groups for assignation in study arms (A-single dose vaccination or B-two doses vaccination). The language has been adjusted (lines 104 and 107)

	and then randomly selected within each age group, or if a different method was used?	
2	In the Methods section of the abstract, the authors mention measuring vibriocidal antibody titers on days 4–6 post-vaccination. However, vibriocidal titers typically peak around day 10 after oral cholera vaccination (see: https://www.sciencedirect.com/science/article/abs/pii/0264410X94903352). Could the authors justify why an earlier time point was chosen and discuss how this may have affected the observed immune response?	We understand that peak vibriocidal titers peak around day 10; however, we were using the early reponse as an indicator of a booster response as had been suggested,<br>We also measured titers at the more typical points when one would expect to see a peak response.<br><br>.
3	It is unclear why the vibriocidal titers were measured at different time points post-vaccination in groups A and B. Could the authors provide a clearer rationale for the differences in sampling schedules across groups?	The day zero titer established the baseline titer for both groups.<br>The early time point for group A was to find evidence of a booster response. For group B, we obtained a similar serum sample early after the second dose. The later sera were collected to understand the persistence of the titers in the groups.
4	The manuscript provides limited information on the logistic regression analyses. Could the authors specify how the linearity assumption was assessed? Additionally, more detail is needed on the covariates included—what specific "household characteristics" were analyzed, and how were these variables selected for inclusion in the model?	Thanks for this comment, to test the linearity of the quantitative variable of the model, we ran a logit regression with quantitative variable (weight and height) in the quadratic form, we found that there is a linear relationship between Seroconversion rate and the quantitative variable of how model.<br>The list of variables used has been described under each table and these variables have been select for two reasons. First, they were a significant relation between these variables and the belonging to study groups (Table 2) and secondly based on literature (DOI: 10.1016/S2214-109X(14)70368-7).
5	How many participants had vaccination status confirmed by vaccination cards versus self-report? In many Sub-Saharan African settings where oral vaccines are given regularly, recall bias—especially four years post-vaccination—can be substantial. Would the authors consider	We enrolled 136 participants with 0 dose, 101 who had received one dose OCV with status tracked, 106 who had received two OCV doses with status tracked, and 7 who received two OCV doses with vaccination card.<br>The number of participants with proof of vaccination on their vaccination card was limited to those with two doses and reduced to 07 participants. We cannot perform an analysis restricted to a single type of exposure to vaccination.

	performing a sensitivity analysis restricted to participants with card-confirmed vaccination status? This could strengthen the internal validity of the findings and might even be presented as the primary analysis.	OCV, being an oral vaccine given to the entire age spectrum is quite different from other injectable vaccines which hare given to children. Obviously, it would have been optimal to have had proof of vaccination, but this was not possible in this case.
6	Could repeated, low-grade or undetected natural exposure to Vibrio cholerae in the study setting have reduced the differences in vibriocidal antibody responses between vaccinated and unvaccinated participants? This potential confounder may warrant discussion in the manuscript.	Following the cholera vaccination campaign organized in 2017, our team conducted a year-long epidemiological surveillance of cholera, screening suspected cases using rapid diagnostic tests for cholera. During this surveillance period, no positive case was detected. It is therefore unlikely that low-grade or undetected natural exposure to Vibrio cholerae in the study setting have reduced the differences in vibriocidal antibody responses between vaccinated and unvaccinated participants. This has been added into discussion: line 384-389.
	Reviewer 1	
	This is an interesting study, addressing important questions. However the study design suggests that its an RCT, however the title does not reflect this and is therefore misleading and so should be updated.<br><br>As this reported as randomised controlled trial - the manuscript should follow CONSORT reporting guidelines, as they are some fundamental methodological elements not reported in the manuscript.	The title has been updated.<br><br>The manuscript follows the CONSORT reporting as it includes in the methods and results part key elements CONSORT reporting points (description of the population, intervention, control, outcome assessment, randomization approach and the CONSORT flow diagram.
	Sample size section- can this be written so that its clear the total number of participants required, its a difference in proportion between control and intervention, and so should be presented to reflect that and also inflated for the drop-out rate. Because earlier there is mention to two stratification factors age and exposure status. In the abstract a	To determine whether a rapid vibriocidal response following single dose OCV administration among those exposed to OCV four years earlier is superior to those exposed to zero doses four years earlier, we attempted to recruit 20 people for each age group (1-4, 5-14, ≥15 years old) in group A and the same number for group B. Among those who had received OCV four years earlier, we had hoped to divide these people into a group receiving one dose and two doses earlier. Thus, total sample size was estimated to be 360. We hypothesized that an early vibriocidal response will occur among 25% of those in control group (those exposed to zero OCV dose four years earlier) and will be increased to 70% in the intervention

	number of 350 is given but 430 were approached?<br>Note that stratification factors are considered to ensure balance between group, i.e control and intervention.	group (those exposed to OCV doses four years earlier (confidence=95%, power=90%), and that the dropout rate in each age group will be 10%.
	Randomization - how was allocation concealment accounted for. Since the block size is 2 - which really should have been variables, i.e 2, 4 to discourage predictability.	Randomization was stratified by age group and prior exposure status to OCV. Thus, in the stratum of each age group, participants who had received zero, one or two doses of OCV were randomly assigned in blocks of two to Arm A or Arm B. The team responsible for randomization was different from the team enrolling and randomly assigning participants to study arms. The randomization procedure provided to generate the four possible combinations of blocks of two (AA, AB, BA, BB, each combination indicating the assignment of each pair of participants to the two study arms) and, to rank per stratum the 180 blocks of expected 360 participants in random order and associated with numbers to generate unique code that indicted the study arm of each participant. The code was printed on a label, inserted into opaque envelopes arranged per stratum in order and numbered. The randomization was implemented by taking per stratum (age group and vaccination status) an envelope for an included patient following the order of numbers dictated by the randomization team, opening it, removing the label and assigning the number to the participant.<br>The procedure was developed to significantly limit chances of predictability.
	What was the randomisation system used, i.e randomisation schedule used, computer generated and who generated the reandomisation schedule?	The randomization was computer generated. It was done by a team that was not involved in participants enrolment and inclusion.
	What was the process when randomising partcipants - all thes details need to be included in the manuscript? Any blinding, if so who? if not then state if this open label and the rataionale.	The procedure of randomization is inserted in the randomization section.
	Statistical analysis section requires more information:	Details regarding logistic regression made for data analysis have been added in the data analysis section from line 172.

	- Define population for analysis<br>- Any indication of how missing data would be handled?<br>- Primary analysis, with the number of participants enrolled, doing a simple t-test is not best approach as the analysis is not accounting/adjusting for the stratification factors - can authors perform some regression analysis - its mentioned further on in the stats section - but still unclear.<br>-Same comments for secondary analyses	
	Results:<br><br>Table 1 - as this randomised controlled study - not advisable to test for differences as baseline, as any differences observed would be by chance - so advise to omit p-value column and updated stats section accordingly.<br>Table 1 - keep use of terms consistent in the report, i.e Arm A, Cohort A ?<br><br>Table 1 - Age median(mean) - usually presented as Age - Median (IQR), OR Age - Mean (SD) - certainly present both.<br>For the variables in table - not expecting to see CI, either range, IQR or SD should be given - please update.<br>Equally for Table 2, where these comparison pre-specified, suggested to keep as descriptive analyses,<br><br>The main comparisons should ideally be Arm A vs Arm B.	Cohort was revised to Arm throughout the manuscript.<br>Thanks for the commence. P.value has been removed from table 1 and 2<br>Table 1 has been updated by using IQR when presenting Age Median

	The authors should really excerse caution with so many tests - that may not have been necessarity prespecified and little numbers, most results should be limited to descriptive analyses.	
	**Reviewer 2**	
	In this manuscript, the authors aim to compare vibriocidal responses among individuals during a re-vaccination campaign conducted 4 years following a previous campaign in Cameroon.<br><br>This is an important and carefully designed study. However, the manuscript requires substantial revision to improve clarity and accuracy. The authors also need to revisit their conclusions in light of the results.<br><br>The methods and results sections contain numerous inconsistencies in numbers and study design elements (such as unclear references to what Arms A and B represent). Additionally, much of the methods and results sections are difficult to follow. The manuscript needs to be rewritten with particular attention to clearly describing comparisons and findings for each result, especially given the study's complex design. Below, I have detailed specific instances of these issues, along with broader comments regarding the conclusions that must be addressed before publication.	
1	The study was designed to compare age-stratified rapid vibriocidal responses following OCV between individuals who had been vaccinated 4 years	Thank you for these comments. We have adjusted the conclusion accordingly.

	earlier and those who had not. The researchers found no significant difference between these groups. However, this finding alone does not support the authors' conclusion that two doses are required for re-vaccination campaigns (abstract lines 46-47). This is particularly true given that they did observe significantly increased vibriocidal responses for single-dose revaccination at day 9, and considering that rapid vibriocidal responses have not been established as a correlate of protection.	
	Does Arm A and B refer to receipt of 1 or 2 doses in the past (as described in the results lines 252-253) or in the present study (as described in the methods and Figure 2)? This is crucial information that must be clarified consistently throughout the manuscript.	Arm A refers to receipt of one dose at day 0 in the present trial, while Arm B refers to receipt of 2 doses at day 0 and day 14 respectively in the present trial. This is explained in the methods section (line 104-105).
3	If Arms A and B correspond to what is described in the methods and Figure 2, are the comparisons between these arms shown in Table 5 and Figure 5? Please clarify this relationship. If so, the results suggest that participants did not exhibit a stronger vibriocidal response following two-dose re-vaccination compared to one-dose re-vaccination. This finding does not support the conclusion stated in the abstract that two doses are needed for re-vaccination campaigns.	We agree with your comments and have drawn a conclusion.
4	The full manuscript would benefit from careful copyediting. For example, lines 31-34 in the	Some selected sentences including lined 31-34 have been edited.

	abstract are difficult to follow, and could be broken up into two sentences.	
5	The results section in the abstract is also difficult to follow and should be revised. This section should also include confidence intervals and statistical tests conducted.	The drafting of the results part of the abstract has been revised, thank you.
6	Line 35 and line 41 in the abstract. Suggest including the specific time intervals instead of “periodically” and “earlier”, respectively.	This was done (35-36 and line 42)
7	Line 80 in the introduction should clarify that the youngest age group examined is 4-year-olds, not 1–4-year-olds, as children under 4 were excluded from the study according to line 96.	This has been revised.
	Line 100. “Samples were collected periodically”. Please include the specific time intervals.	The specific time intervals were added.
	Line 116 states that individuals 1 and older were included, but this is inconsistent with the study design, where it stated that individuals 4 and older were included.	This has been revised
	In the sample size section, please clarify how sample size calculations considered Arm A and B of the intervention. As written, only Arm A is described.	The drafting of the sample size estimate process has been revised. See from line 131.
	• In Table 1, please clarify if Cohort A and Cohort B are the same as Arm A and Arm B. Based on Figure 3, is appears and Cohort A and B refer to people who did and did not received the vaccine previously, which is confusing as Arm A and B refer to those that received 1 or 2 doses in this study. This needs to	Cohort in the result section stood for Arm (Arm A which correspond to 1 dose administered and Arm to 2 doses administered), this was corrected and harmonized throughout the manuscript.

	be made very clear in the manuscript.	
	All figures require more detailed legends.	We have provided detail legend where needed.
	Lines 263-267. These sentences are very general. Please be explicit in what was found.	We have revised the sentences.
	What do Arm A and Arm B refer to in Figure 5? Please also include a more detailed description of these results in the text on lines 307-308.	Arm A refer to study participants who receive one OCV vaccine dose at day 0 of the study and Arm B refer to study participants who received two OCV doses at days 0 and 14 of the study.
	**Reviewer 3**	
1	Although the manuscript notes that the study intervention (OCV) was administered four years after prior vaccination, it would enhance clarity to explicitly state the actual study period (i.e., study start and end dates).	Enrollment of participants for the present trial was conducted from February to June 2021. This is mentioned in setting (line115) and “Enrollment” (Line 164).
2	In Figure 1, it is unclear whether the first blood draw was conducted prior to vaccination (as a baseline) or after vaccination. Clarifying this would help the reader interpret the immunogenicity results appropriately.	The first blood draw was conducted prior to the first vaccine dose administration.
3	It appears that adverse events were reported during the study period. If such data are available, including the conclusion drawn from those safety data, would strengthen the manuscript by providing insight into the safety of administering additional OCV doses in individuals with prior vaccination history.	. Detected AEFI are described in line 354-357
4	In the baseline characteristics, variables such as "cement/brick/tiles" and "use of improved toilet facilities" may be context-specific. A brief explanation of the relevance of	These are variables describing the participant's socioeconomic status through characteristics of their household and access to WASH.

	these indicators would help for clarity.	
5	In this clinical trial, the procedures involved multiple blood draws and administration of OCV, which may have contributed to protocol deviations. If a per-protocol set (PPS) was pre-defined, it would be informative to include the number of participants in the PPS in the CONSORT diagram, especially if it differs substantially from the full analysis set.	This is specified in the CONSORT diagram
6	It might be helpful to also provide GMT without log-transformation in the table as seen in other OCV publications.	Thanks for this comment,<br>table A1, A2 and A3 with the value of GMT of each group has been added in annex.
7	The discussion mentions malnutrition as a possible contributor to immune response variability. If heights were collected along with the weight data provided, it would be helpful to include BMI in the demographic table to better support this interpretation.	Thanks for the comment,<br>BMI has been added into the demographic tables.
8	Regarding the study's conclusions about revaccination with one versus two doses, the following limitations would need to be acknowledged:<br><br>a) The sample size seems relatively small, particularly within certain age strata.<br><br>b) The study focuses solely on short-term immunogenicity outcomes without evaluating clinical efficacy against cholera, which warrants caution when interpreting the findings in the context of public health policy and vaccine recommendations.	The limitations have been acknowledged

	Reviewer 4	
1	Team has written an important manuscript on oral cholera vaccine. Spacing is an important issue that policy makers need to revisit for OCV. Therefore, such type of research should come out quickly to know the picture of OCV. Few comments that need to be discussed in the manuscript.	-
2	Why authors choose 4 years interval?	We planned to conduct the intervention assessment in participants vaccinated three years earlier. This the time documented to have OCV vaccine efficacy and effectiveness dropping at 25% or less. As specified in this study : https://www.thelancet.com/journals/langlo/article/PIIS2214-109X(25)00107-X/fulltext But the COVID 19 pandemic negative effect on vaccines supply from importers, the vaccines were received one year later than planned. This is why the intervention was assessed in a population vaccinated four years earlier.
3	Is there any differences in the vibriocidal titer in different age group after 4 years vaccination?	This was not part of our data analysis plan.
4	When OCV production is really challenging to serve the demand even for a single dose, how feasible to think about double dose?	Although the ICG is allowing only for a single dose for emergency use of vaccine (due to vaccine shortage), the recommendation remains that this should be a two-dose vaccine. Once the supply is improved, it is expected that two doses will be used for preventive campaigns since this is the dose schedule that has been documented to be effective and is crucial to protect children under age 5.
