# Supplementary material for: Serological responses to killed oral cholera vaccine (OCV) when given 4 years after initial receipt of OCV in Cameroon: A randomized controlled trial
Source: PLOS Glob Public Health. 2026 Apr 21;6(4):e0004913. doi: 10.1371/journal.pgph.0004913 (PMC13098963; doi:10.1371/journal.pgph.0004913)
Supplement: Attachment — Submitted filename: Response to Reviewers(2).docx [file pgph.0004913.s006.docx]

Response to Reviewers

PGPH-D-25-01336

Serological responses to killed oral cholera vaccine (OCV) when given 4 years after initial receipt of OCV in Cameroon

|  | **Comments** | **Responses** |
| --- | --- | --- |
|  | **Journal Requirement** |  |
| 1 | Please provide additional details regarding participant consent. In the ethics statement in the Methods and online submission information, please ensure that you have specified (1) whether consent was informed and (2) what type you obtained (for instance, written or verbal, and if verbal, how it was documented and witnessed). If your study included minors, state whether you obtained consent from parents or guardians. If the need for consent was waived by the ethics committee, please include this information.  If you are reporting a retrospective study of medical records or archived samples, please ensure that you have discussed whether all data were fully anonymized before you accessed them and/or whether the IRB or ethics committee waived the requirement for informed consent. If patients provided informed written consent to have data from their medical records used in research, please include this information. | The methods section of the manuscript includes an ethical consideration section that gives all details about the consent process during the study. Informed and written consent was obtained for all included participants. For participants aged below 12, parental permission was obtained. For participants aged 12-20 years, the assent was obtained from the participant accompanied by a parental/guardian permission. For individuals aged 20 years and above, written consent was obtained from the participant. This is documented from line 258 to 265.  We are not reporting retrospective study of medical records or archived samples. |
| 2 | We are unable to open your Supporting Information file “Base MOBS treated.dta”. Please kindly revise as necessary and re-upload. | We uploaded a new version of the database. The previous version of the database was made using Stata18, hence the difficulty to open with other versions of the software. To avoid this, we saved the data in the way that the old version of Stata will be able to open. |
| 3 | Please provide separate figure files in .tif or .eps format. | New versions of the figures have been uploaded in the indicated formats. |
| 4 | We have amended your Competing Interest statement to comply with journal style. We kindly ask that you double check the statement and let us know if anything is incorrect. | We approve the amended version. |
| 5 | Your current Financial Disclosure states, “This work was supported by the Department for International Development and Wellcome Trust: grant number: 215663/Z/19/Z and National Institute of Allergy and Infectious Disease (5R01AI123422)”. However, your funding information on the submission form indicates that you received funding from “Wellcome Trust” with grant number “215663/Z/19/Z”. Please indicate by return email the full and correct funding information for your study and confirm the order in which funding contributions should appear. Please be sure to indicate whether the funders played any role in the study design, data collection and analysis, decision to publish, or preparation of the manuscript. | This work was supported by a grant from the Wellcome Trust to JA (grant number: 215663/Z/19/Z) and a grant from the National Institute of Allergy and Infectious Disease to DAS (5R01AI123422). The Wellcome Trust provided suggestions as to the study design, but neither funder had any role in data collection and analysis, decision to publish, or preparation of the manuscript.  This was adjusted in the manuscript from line 444-447. |
| 6 | Some material included in your submission may be copyrighted. According to PLOS’s copyright policy, authors who use figures or other material (e.g., graphics, clipart, maps) from another author or copyright holder must demonstrate or obtain permission to publish this material under the Creative Commons Attribution 4.0 International (CC BY 4.0) License used by PLOS journals. Please closely review the details of PLOS’s copyright requirements here: PLOS Licenses and Copyright. If you need to request permissions from a copyright holder, you may use PLOS's Copyright Content Permission form.  Please respond directly to this email or email the journal office and provide any known details concerning your material's license terms and permissions required for reuse, even if you have not yet obtained copyright permissions or are unsure of your material's copyright compatibility.  Potential Copyright Issues: Figure 1: please (a) provide a direct link to the base layer of the map (i.e., the country or region border shape) and ensure this is also included in the figure legend; and (b) provide a link to the terms of use / license information for the base layer image or shapefile. We cannot publish proprietary or copyrighted maps (e.g. Google Maps, Mapquest) and the terms of use for your map base layer must be compatible with our CC-BY 4.0 license.  Note: if you created the map in a software program like R or ArcGIS, please locate and indicate the source of the basemap shapefile onto which data has been plotted. | The map of figure 1 was created using the software QGIS 2.18. The base map shapefile and layers were  obtained from the Health Information Unit of the Cameroon  Ministry of Public Health which is provided upon request. The figure can be deleted in case it still represents an issue. |
| 7 | If the reviewer comments include a recommendation to cite specific previously published works, please review and evaluate these publications to determine whether they are relevant and should be cited. There is no requirement to cite these works unless the editor has indicated otherwise. | There was no recommendation requesting to cite specific previously published work. |
|  | **Additional comments** |  |
| 1 | Line 98: The phrase “randomly stratified by age” appears ambiguous. Stratification and randomization are typically distinct steps in study design. Could the authors clarify whether participants were stratified by age and then randomly selected within each age group, or if a different method was used? | Participants were organized in stratus of age groups (1-4 year, 5-14, ≥15 years old) and randomization was done independently in each of the age groups for assignation in study arms (A-single dose vaccination or B-two doses vaccination). The language has been adjusted (lines 104 and 107) |
| 2 | In the Methods section of the abstract, the authors mention measuring vibriocidal antibody titers on days 4–6 post-vaccination. However, vibriocidal titers typically peak around day 10 after oral cholera vaccination (see:  https://www.sciencedirect.com/science/article/abs/pii/0264410X94903352 ). Could the authors justify why an earlier time point was chosen and discuss how this may have affected the observed immune response? | We understand that peak vibriocidal titers peak around day 10; however, we were using the early reponse as an indicator of a booster response as had been suggested,  We also measured titers at the more typical points when one would expect to see a peak response.  . |
| 3 | It is unclear why the vibriocidal titers were measured at different time points post-vaccination in groups A and B. Could the authors provide a clearer rationale for the differences in sampling schedules across groups? | The day zero titer established the baseline titer for both groups.  The early time point for group A was to find evidence of a booster response. For group B, we obtained a similar serum sample early after the second dose. The later sera were collected to understand the persistence of the titers in the groups. |
| 4 | The manuscript provides limited information on the logistic regression analyses. Could the authors specify how the linearity assumption was assessed? Additionally, more detail is needed on the covariates included—what specific “household characteristics” were analyzed, and how were these variables selected for inclusion in the model? | Thanks for this comment, to test the linearity of the quantitative variable of the model, we ran a logit regression with quantitative variable (weight and height) in the quadratic form, we found that there is a linear relationship between Seroconversion rate and the quantitative variable of how model.  The list of variables used has been described under each table and these variables have been select for two reasons. First, they were a significant relation between these variables and the belonging to study groups (Table 2) and secondly based on literature (DOI: 10.1016/S2214-109X(14)70368-7). |
| 5 | How many participants had vaccination status confirmed by vaccination cards versus self-report? In many Sub-Saharan African settings where oral vaccines are given regularly, recall bias—especially four years post-vaccination—can be substantial. Would the authors consider performing a sensitivity analysis restricted to participants with card-confirmed vaccination status? This could strengthen the internal validity of the findings and might even be presented as the primary analysis. | We enrolled 136 participants with 0 dose, 101 who had received one dose OCV with status tracked, 106 who had received two OCV doses with status tracked, and 7 who received two OCV doses with vaccination card.  The number of participants with proof of vaccination on their vaccination card was limited to those with two doses and reduced to 07 participants. We cannot perform an analysis restricted to a single type of exposure to vaccination.  OCV, being an oral vaccine given to the entire age spectrum is quite different from other injectable vaccines which hare given to children. Obviously, it would have been optimal to have had proof of vaccination, but this was not possible in this case. |
| 6 | Could repeated, low-grade or undetected natural exposure to Vibrio cholerae in the study setting have reduced the differences in vibriocidal antibody responses between vaccinated and unvaccinated participants? This potential confounder may warrant discussion in the manuscript. | Following the cholera vaccination campaign organized in 2017, our team conducted a year-long epidemiological surveillance of cholera, screening suspected cases using rapid diagnostic tests for cholera. During this surveillance period, no positive case was detected. It is therefore unlikely that low-grade or undetected natural exposure to Vibrio cholerae in the study setting have reduced the differences in vibriocidal antibody responses between vaccinated and unvaccinated participants. This has been added into discussion: line 384-389. |
|  | Reviewer 1 |  |
|  | This is an interesting study, addressing important questions. However the study design suggests that its an RCT, however the title does not reflect this and is therefore misleading and so should be updated.  As this reported as randomised controlled trial - the manuscript should follow CONSORT reporting guidelines, as they are some fundamental methodological elements not reported in the manuscript. | The title has been updated.  The manuscript follows the CONSORT reporting as it includes in the methods and results part key elements CONSORT reporting points (description of the population, intervention, control, outcome assessment, randomization approach and the CONSORT flow diagram. |
|  | Sample size section- can this be written so that its clear the total number of participants required, its a difference in proportion between control and intervention, and so should be presented to reflect that and also inflated for the drop-out rate. Because earlier there is mention to two stratification factors age and exposure status. In the abstract a number of 350 is given but 430 were approached?  Note that stratification factors are considered to ensure balance between group, i.e control and intervention. | To determine whether a rapid vibriocidal response following single dose OCV administration among those exposed to OCV four years earlier is superior to those exposed to zero doses four years earlier, we attempted to recruit 20 people for each age group (1-4, 5-14, ≥15 years old) in group A and the same number for group B. Among those who had received OCV four years earlier, we had hoped to divide these people into a group receiving one dose and two doses earlier. Thus, total sample size was estimated to be 360. We hypothesized that an early vibriocidal response will occur among 25% of those in control group (those exposed to zero OCV dose four years earlier) and will be increased to 70% in the intervention group (those exposed to OCV doses four years earlier (confidence=95%, power=90%), and that the dropout rate in each age group will be 10%. |
|  |  |  |
|  | Randomization - how was allocation concealment accounted for. Since the block size is 2 - which really should have been variables, i.e 2, 4 to discourage predictability. | Randomization was stratified by age group and prior exposure status to OCV. Thus, in the stratum of each age group, participants who had received zero, one or two doses of OCV were randomly assigned in blocks of two to Arm A or Arm B. The team responsible for randomization was different from the team enrolling and randomly assigning participants to study arms. The randomization procedure provided to generate the four possible combinations of blocks of two (AA, AB, BA, BB, each combination indicating the assignment of each pair of participants to the two study arms) and, to rank per stratum the 180 blocks of expected 360 participants in random order and associated with numbers to generate unique code that indicted the study arm of each participant. The code was printed on a label, inserted into opaque envelopes arranged per stratum in order and numbered. The randomization was implemented by taking per stratum (age group and vaccination status) an envelope for an included patient following the order of numbers dictated by the randomization team, opening it, removing the label and assigning the number to the participant.  The procedure was developed to significantly limit chances of predictability. |
|  | What was the randomisation system used, i.e randomisation schedule used, computer generated and who generated the reandomisation schedule? | The randomization was computer generated. It was done by a team that was not involved in participants enrolment and inclusion. |
|  | What was the process when randomising partcipants - all thes details need to be included in the manuscript? Any blinding, if so who? if not then state if this open label and the rataionale. | The procedure of randomization is inserted in the randomization section. |
|  | Statistical analysis section requires more information:  - Define population for analysis  - Any indication of how missing data would be handled?  - Primary analysis, with the number of participants enrolled, doing a simple t-test is not best approach as the analysis is not accounting/adjusting for the stratification factors - can authors perform some regression analysis - its mentioned further on in the stats section - but still unclear.  -Same comments for secondary analyses | Details regarding logistic regression made for data analysis have been added in the data analysis section from line 172. |
|  | Results:  Table 1 - as this randomised controlled study - not advisable to test for differences as baseline, as any differences observed would be by chance - so advise to omit p-value column and updated stats section accordingly.  Table 1 - keep use of terms consistent in the report, i.e Arm A, Cohort A ?  Table 1 - Age median(mean) - usually presented as Age - Median (IQR), OR Age - Mean (SD) - certainly present both.  For the variables in table - not expecting to see CI, either range, IQR or SD should be given - please update.  Equally for Table 2, where these comparison pre-specified, suggested to keep as descriptive analyses,  The main comparisons should ideally be Arm A vs Arm B.  The authors should really excerse caution with so many tests - that may not have been necessarity prespecified and little numbers, most results should be limited to descriptive analyses. | Cohort was revised to Arm throughout the manuscript.  Thanks for the commence. P.value has been removed from table 1 and 2  Table 1 has been updated by using IQR when presenting Age Median |
|  | **Reviewer 2** |  |
|  | In this manuscript, the authors aim to compare vibriocidal responses among individuals during a re-vaccination campaign conducted 4 years following a previous campaign in Cameroon.  This is an important and carefully designed study. However, the manuscript requires substantial revision to improve clarity and accuracy. The authors also need to revisit their conclusions in light of the results.  The methods and results sections contain numerous inconsistencies in numbers and study design elements (such as unclear references to what Arms A and B represent). Additionally, much of the methods and results sections are difficult to follow. The manuscript needs to be rewritten with particular attention to clearly describing comparisons and findings for each result, especially given the study's complex design. Below, I have detailed specific instances of these issues, along with broader comments regarding the conclusions that must be addressed before publication. |  |
| 1 | The study was designed to compare age-stratified rapid vibriocidal responses following OCV between individuals who had been vaccinated 4 years earlier and those who had not. The researchers found no significant difference between these groups. However, this finding alone does not support the authors' conclusion that two doses are required for re-vaccination campaigns (abstract lines 46-47). This is particularly true given that they did observe significantly increased vibriocidal responses for single-dose revaccination at day 9, and considering that rapid vibriocidal responses have not been established as a correlate of protection. | Thank you for these comments. We have adjusted the conclusion accordingly. |
|  | Does Arm A and B refer to receipt of 1 or 2 doses in the past (as described in the results lines 252-253) or in the present study (as described in the methods and Figure 2)? This is crucial information that must be clarified consistently throughout the manuscript. | Arm A refers to receipt of one dose at day 0 in the present trial, while Arm B refers to receipt of 2 doses at day 0 and day 14 respectively in the present trial. This is explained in the methods section (line 104-105). |
| 3 | If Arms A and B correspond to what is described in the methods and Figure 2, are the comparisons between these arms shown in Table 5 and Figure 5? Please clarify this relationship. If so, the results suggest that participants did not exhibit a stronger vibriocidal response following two-dose re-vaccination compared to one-dose re-vaccination. This finding does not support the conclusion stated in the abstract that two doses are needed for re-vaccination campaigns. | We agree with your comments and have drawn a conclusion. |
| 4 | The full manuscript would benefit from careful copyediting. For example, lines 31-34 in the abstract are difficult to follow, and could be broken up into two sentences. | Some selected sentences including lined 31-34 have been edited. |
| 5 | The results section in the abstract is also difficult to follow and should be revised. This section should also include confidence intervals and statistical tests conducted. | The drafting of the results part of the abstract has been revised, thank you. |
| 6 | Line 35 and line 41 in the abstract. Suggest including the specific time intervals instead of “periodically” and “earlier”, respectively. | This was done (35-36 and line 42) |
| 7 | Line 80 in the introduction should clarify that the youngest age group examined is 4-year-olds, not 1–4-year-olds, as children under 4 were excluded from the study according to line 96. | This has been revised. |
|  | Line 100. “Samples were collected periodically”. Please include the specific time intervals. | The specific time intervals were added. |
|  | Line 116 states that individuals 1 and older were included, but this is inconsistent with the study design, where it stated that individuals 4 and older were included. | This has been revised |
|  | In the sample size section, please clarify how sample size calculations considered Arm A and B of the intervention. As written, only Arm A is described. | The drafting of the sample size estimate process has been revised. See from line 131. |
|  | • In Table 1, please clarify if Cohort A and Cohort B are the same as Arm A and Arm B. Based on Figure 3, is appears and Cohort A and B refer to people who did and did not received the vaccine previously, which is confusing as Arm A and B refer to those that received 1 or 2 doses in this study. This needs to be made very clear in the manuscript. | Cohort in the result section stood for Arm (Arm A which correspond to 1 dose administered and Arm to 2 doses administered), this was corrected and harmonized throughout the manuscript. |
|  | All figures require more detailed legends. | We have provided detail legend where needed. |
|  | Lines 263-267. These sentences are very general. Please be explicit in what was found. | We have revised the sentences. |
|  | What do Arm A and Arm B refer to in Figure 5? Please also include a more detailed description of these results in the text on lines 307-308. | Arm A refer to study participants who receive one OCV vaccine dose at day 0 of the study and Arm B refer to study participants who received two OCV doses at days 0 and 14 of the study. |
|  | **Reviewer 3** |  |
| 1 | Although the manuscript notes that the study intervention (OCV) was administered four years after prior vaccination, it would enhance clarity to explicitly state the actual study period (i.e., study start and end dates). | Enrollment of participants for the present trial was conducted from February to June 2021. This is mentioned in setting (line115) and “Enrollment” (Line 164). |
| 2 | In Figure 1, it is unclear whether the first blood draw was conducted prior to vaccination (as a baseline) or after vaccination. Clarifying this would help the reader interpret the immunogenicity results appropriately. | The first blood draw was conducted prior to the first vaccine dose administration. |
| 3 | It appears that adverse events were reported during the study period. If such data are available, including the conclusion drawn from those safety data, would strengthen the manuscript by providing insight into the safety of administering additional OCV doses in individuals with prior vaccination history. | . Detected AEFI are described in line 354-357 |
| 4 | In the baseline characteristics, variables such as "cement/brick/tiles" and "use of improved toilet facilities" may be context-specific. A brief explanation of the relevance of these indicators would help for clarity. | These are variables describing the participant's socioeconomic status through characteristics of their household and access to WASH. |
| 5 | In this clinical trial, the procedures involved multiple blood draws and administration of OCV, which may have contributed to protocol deviations. If a per-protocol set (PPS) was pre-defined, it would be informative to include the number of participants in the PPS in the CONSORT diagram, especially if it differs substantially from the full analysis set. | This is specified in the CONSORT diagram |
| 6 | It might be helpful to also provide GMT without log-transformation in the table as seen in other OCV publications. | Thanks for this comment,  table A1, A2 and A3 with the value of GMT of each group has been added in annex. |
| 7 | The discussion mentions malnutrition as a possible contributor to immune response variability. If heights were collected along with the weight data provided, it would be helpful to include BMI in the demographic table to better support this interpretation. | Thanks for the comment,  BMI has been added into the demographic tables. |
| 8 | Regarding the study’s conclusions about revaccination with one versus two doses, the following limitations would need to be acknowledged:  a) The sample size seems relatively small, particularly within certain age strata.  b) The study focuses solely on short-term immunogenicity outcomes without evaluating clinical efficacy against cholera, which warrants caution when interpreting the findings in the context of public health policy and vaccine recommendations. | The limitations have been acknowledged |
|  | **Reviewer 4** |  |
| 1 | Team has written an important manuscript on oral cholera vaccine. Spacing is an important issue that policy makers need to revisit for OCV. Therefore, such type of research should come out quickly to know the picture of OCV. Few comments that need to be discussed in the manuscript. | - |
| 2 | Why authors choose 4 years interval? | We planned to conduct the intervention assessment in participants vaccinated three years earlier. This the time documented to have OCV vaccine efficacy and effectiveness dropping at 25% or less. As specified in this study : <https://www.thelancet.com/journals/langlo/article/PIIS2214-109X(25)00107-X/fulltext>  But the COVID 19 pandemic negative effect on vaccines supply from importers, the vaccines were received one year later than planned. This is why the intervention was assessed in a population vaccinated four years earlier. |
| 3 | Is there any differences in the vibriocidal titer in different age group after 4 years vaccination? | This was not part of our data analysis plan. |
| 4 | When OCV production is really challenging to serve the demand even for a single dose, how feasible to think about double dose? | Although the ICG is allowing only for a single dose for emergency use of vaccine (due to vaccine shortage), the recommendation remains that this should be a two-dose vaccine. Once the supply is improved, it is expected that two doses will be used for preventive campaigns since this is the dose schedule that has been documented to be effective and is crucial to protect children under age 5. |
