# Supplementary material for: Serological responses to killed oral cholera vaccine (OCV) when given 4 years after initial receipt of OCV in Cameroon: A randomized controlled trial
Source: PLOS Glob Public Health. 2026 Apr 21;6(4):e0004913. doi: 10.1371/journal.pgph.0004913 (PMC13098963; doi:10.1371/journal.pgph.0004913)
Supplement: Attachment — Submitted filename: Response to comments march20.docx [file pgph.0004913.s008.docx]

**Response to comments**

Title: Serological responses to killed oral cholera vaccine (OCV) when given 4 years after initial receipt of OCV in Cameroon: A randomized controlled trial

| Comments | Responses |
| --- | --- |
| Thank you for submitting your revised manuscript. We appreciate the effort invested in responding to the reviewers’ comments. However, after careful evaluation of the revised version and the authors’ responses, we conclude that the manuscript still requires substantial improvement before it can be considered for acceptance. A major revision is therefore required. In addition to the comments from the reviewers, we outline below the main issues that must be addressed to improve clarity, methodological rigor, and interpretability of the findings. | The response to editor and reviewers’ comments are presented below. |
| Abstract • The background section of the abstract is overly long, in places more detailed than the results section. Please streamline this section. | The abstract has been revised and the number of words reduced. |
| The conclusion of the abstract contains French-influenced expressions and requires correction. | This was revised. |
| • The abstract conclusion should be strengthened. Rather than restating results, the authors should explain what the findings imply in practical and programmatic terms, particularly in relation to the issue of OCV revaccination raised in the background. | The implications of the results were added. |
| • Overall, the manuscript would benefit from thorough proofreading, particularly to improve grammar, punctuation, and phrasing. | The manuscript was revised accordingly, thank you. |
| Background |  |
| The first sentences are too generic and non-informative.  Lines 55-57: The term “high case-fatality rate” is vague. Could the authors provide some data (numbers) to support the claims here? In many endemic settings, cholera case-fatality rates have declined substantially over the past decades due to improvements in rehydration therapy. Please clarify what is meant here and ensure it is supported by the cited references. The statement regarding the “negative impact on the performance of health systems” is unclear. It is not evident whether the cited references directly assessed health system performance. Please clarify or rephrase. | The first paragraph of the introduction has been reformulated to improve the readability and content. Modifications can be consulted from line 54-60. |
| The paragraph spanning lines 66–89 is overly long and should be split into at least two paragraphs for improved readability. | Thank you, this was revised accordingly. |
| Lines 90 to 94: the text is presented as a study protocol for a study that will be conducted later. The authors could consider reformulating it. | The paragraph was revised (line 94-95). |
| Methods 1. Ascertainment of vaccination status • The authors indicate that only 7 participants had vaccination confirmed by card, meaning vaccination status was largely self-reported. It is unclear how OCV was differentiated from other vaccines administered through routine or campaign activities. Given the 4-year interval since vaccination, recall bias and confusion, particularly among children in LMICs exposed to multiple oral vaccines, are important concerns. These issues must be clearly described in the Methods section and acknowledged as limitations. | The vaccination status of the OCV was tracked using pictures of the vaccine vial and tracking of the vaccination campaign period that was uniquely implemented during the Ramadan season in previous years. This was already described in the methods section (from line 158 to 162). This was added in the limitations (442-444). |
| 2. **Statistical analysis** • The statistical methods section remains poorly written and inconsistent, despite previous editorial and reviewer comments. | The statistical methods section was revised to add more details and correct inconsistencies. |
| • It is unclear whether seroconversion was analyzed as a binary or continuous variable. The manuscript mentions a z-test, while tables refer to chi-square tests. | The seroconversion was analyzed as a binary variable (seroconversion rate) while vibriocidal titer was estimated as a continuous variable. |
| • The methods describe Student’s t-test, but table legends refer to Wilcoxon tests. These inconsistencies must be resolved, and the statistical approach clearly and consistently described. | This was revised and the statistical analysis section modified accordingly. |
| • Details about how the logistic regression was done remain patchy. The description in lines 241–242 is insufficient. | To compare the statistical significance of the vibriocidal seroconversion rate between groups (Arm A and Arm B as well as for other desegregation), we employed a multivariable logistic regression model. The model was used to test the null hypothesis of no difference in seroconversion status (dependent variable) based on vaccination status in 2017 (main independent variable).  To ensure the validity of the p-values, the model adjusted for potential confounding factors at both the individual and household levels, including sex, age group (categorized for all ages), floor material, and access to improved toilet facilities, as well as weight and height. This was added in the data analysis section (line 253-259) |
| Nutritional status Nutritional status is discussed as an important factor, yet its assessment is poorly described in the methods. Please specify: • anthropometric measurements collected, • instruments used, • indices calculated, • and classification criteria applied. • The uniform use of BMI across adults and children is inappropriate. WHO-recommended indices (BMI-for-age for children and adolescents, BMI for adults) should be used. Please revise accordingly.  • If there are data limitations, the authors should explain in the discussion which data were missing. | Anthropometric measurements collected include: height and weight. Detaisl were added in the methodology (line 162-165). The BMI was revised and estimated for participants aged 5 years and above.  Nutritional status classification was not performed as the study was not designed to assess nutritional status as an outcome. |
| Household-level variables included in regression models must be explicitly defined in the Methods, including: • variable definitions, • scales of measurement, • and rationale for inclusion (and if possible references)    6. Wealth, sanitation, and household characteristics • The manuscript includes a limited set of household variables to describe wealth and living conditions, but the rationale for selecting these specific variables is not explained. • Previous studies have shown that in some settings the type of sanitation infrastructure alone does not necessarily predict cholera risk. Rather, sharing status, which affects maintenance, cleanliness, and hygiene practices and is strongly correlated with poverty, has been shown to be a more relevant determinant of cholera transmission. • In this context, poverty and socio-economic status cannot be adequately captured by single variables such as sanitation type or housing material alone. Also, what do the authors mean by housing materials? Do they mean materials for the external walls or materials for the floor? The authors should therefore: • justify why the selected variables are considered the most relevant and sufficient indicators of household wealth and cholera risk; • Explain why other routinely used socio-economic indicators (e.g., electricity in the home, shared sanitation facilities, household crowding, etc.) were not included. • Provide references supporting the validity of their chosen variables in the study setting • This justification should be clearly presented in the Methods section. | We did not find any guidelines specifying the list of variables that must be included to describe socioeconomic indicators and household level variables. During the design phase, we selected socioeconomic indicators that allow us to examine household income levels locally and that may influence the estimation of our indicators.  Household-level socioeconomic and environmental factors were included in the multivariable models to adjust for potential confounding. These variables were defined and categorized as follows:  Floor Material: This was treated as a binary categorical variable (Yes/No). Households with floors constructed of cement, brick, or tiles were categorized as “Yes' (Improved), while all other materials (e.g. earth, sand or dung) were categorized as 'No' (Unimproved).  The Rationale is that floor material is a widely recognized proxy for household socioeconomic status (SES) and domestic hygiene. Improved flooring is associated with reduced transmission of enteric pathogens and better overall health outcomes in low-resource settings (Psaki et al., 2014). https://doi.org/10.1186/1478-7954-12-8  Improved Toilet Facility: This was defined as a binary variable according to the WHO/UNICEF Joint Monitoring Programme (JMP) standards. 'Improved' facilities included flush/pour-flush toilets to piped sewer systems, septic tanks, or pit latrines, and ventilated improved pit (VIP) latrines. the Rationale is that access to sanitation is a critical determinant of cholera exposure risk and may influence the baseline vibriocidal antibody levels of the population. As discussed in the article: <https://doi.org/10.1186/1478-7954-12-8>).  In the study area (Mogode), there is no electricity supply, so almost all households lack access to electricity; therefore, this variable has not been included. This is the same for sanitation facilities, as the majority of households share sanitation facilities. Household crowding was not collected and we agree that it was a limitation. |
| 3. Randomization, p-values, and group imbalance One reviewer suggested removing p-values, noting that participants were randomized to different vaccination regimens. While we understand this rationale, the presence of apparent imbalances between groups (for example, housing materials in Table 2) raises questions regarding the effectiveness of the randomization. It also raises the question of whether including p-values in some tables would be helpful for the reader to better understand these imbalances. The authors should therefore clearly explain: • how they interpret the observed imbalances between groups, • and whether these imbalances are compatible with chance alone or suggest implementation issues. If imbalances exist, the analytical strategy used to account for them should be clearly described in the methods section. | Randomization was stratified according to age group and vaccination thus we do not expect imbalances to be linked to randomization or implementation issues but would rather be attributed to chance. Unbalanced factors were taken into account in the adjustment for the estimation of the main indicators of the study (logistic regression).  The p-values were re-introduced in table 2. |
| Results 1. Table presentation Tables are difficult to interpret, likely due to insufficient methodological clarity. In Table 1, it should be clearly explained why some variables are summarized using median (IQR) while others use mean with 95% CI. If IQRs are used, please specify the 25th and 75th percentiles. The choice of summary statistics must be justified in the Methods section. | The choice between median and mean to summarize the variable is based on the nature of data distribution of the variable. Age, weight and height are not normally distributed so the median was used (25th and 75th percentiles added). VGMT have been normalized using log10, hence mean and CI were used. This was described in the methods (238-239). |
| 2. Table 3 Table 3 is confusing, particularly the final column. The authors state they compare zero doses vs. one or two doses, yet only one column of data is shown. Please clarify whether this column represents descriptive data or a comparison and revise the table accordingly. | The final column presents data on participants that received **at least** one OCV dose, thus a combination of those that received one or two doses.  To avoid redundancy, we did not report the data for zero dose twice in the same table. so, the final p-value column is from the comparison between the zero-dose group (first column with data) and the at least one dose group (fourth column with data). We added clarification as table note. |
| 3. Tables and legends Table legends should be substantially improved. Given that several tables include different types of data within the same table (e.g., descriptive statistics, comparative statistics, regression outputs), the legends must clearly explain what type of data are presented, what comparisons (if any) are being made, and how the data should be interpreted. Methodological details currently embedded in table legends should be moved to the Methods section, while legends should focus on guiding data interpretation. | Summary interpretations of table results are presented in the text preceding the tables. The same indicators are estimated globally for all age groups and presented individually for each targeted age group in the study. Singular statistical tests used to estimate p-values are presented in the table legends. The choice of the statistical tests was revised and presented in the methods section (data analysis line 245-259). Further methodological aspects were moved to the methods. |
| 4. Effect estimates The authors predominantly report p-values, rather than 95% confidence intervals. We strongly recommend reporting 95% CIs, which convey both statistical significance and precision and are generally preferred over p-values alone. | 95% CI were mostly reported throughout the result. P-values are only reported for the comparison between groups. |
| Figures Figure legends should be expanded to allow figures to be interpreted independently. In particular, the authors should clearly specify what the points represent and what the error bars represent (e.g., standard deviation, standard error, or 95% confidence intervals) | Legends of the bars were included in the figures. |
| Conceptual consistency In the Background (lines 86–89), one of the stated research questions concerns whether serum vibriocidal responses differ between children and older age groups. However, in response to Reviewer #4 (comment #3: “Is there any differences in the vibriocidal titer in different age group after 4 years vaccination?”), the authors state that “This was not part of our data analysis plan.” This response is difficult to reconcile with the manuscript text. If the data allow—even in a post hoc analysis—this important question should be addressed. Not doing so would represent a missed opportunity. | We apologize for the previous response addressed to the reviewer number 4; his comment was misunderstood. This was indeed one of the research questions. For each of the indicators analyzed, the results were presented individually per age group targeted (1-4 years, 5-14, ≥15 years). The comparison between children and older participants was introduced in the manuscript (line 344-349, table 5 and figure 6) |
| Missing data: one reviewer asked a question about how the missing data were handled. It is not clear the authors responded to this question. | Throughout the data collection phase, a multi-stage Data Quality Assurance (DQA) process was implemented, including real-time electronic data entry checks and daily supervisory reviews to ensure data completeness.  Due to these measures, the proportion of missing values for the primary outcome and key covariates was minimal. Consequently, we employed a Complete Case Analysis approach. Given the very low drop-out and missingness rates, this approach is expected to yield unbiased estimates without the need for multiple imputation. |
| Discussion Paragraphs 406–412 largely repeat the results and do not provide interpretation. Pages 424-425: “In summary, we had hoped to observe a rapid vibriocidal response to participants who had been vaccinated four years earlier.” It seems the authors are expressing a feeling here. Some might see this as a sign that they were less interested in the truth than in an expected result. Could they rephrase this passage in a more neutral and objective way?  These paragraphs should be revised to offer contextual interpretation, implications, and comparison with existing literature, rather than restating findings. In summary, while the study addresses an important public health question, substantial revisions are required to improve methodological transparency, analytical rigor, and clarity of presentation. We encourage the authors to carefully address each point above and to provide a detailed, point-by-point response with their revised manuscript. | In this paragraph, we summarized the finding that was discussed in the same paragraph and subsequently interpreted in the following lines (line 421-422, 427-436).  The sentence “In summary, we had hoped to observe a rapid vibriocidal response to participants who had been vaccinated four years earlier.” was rephrased (line 445). |
| **Reviewer 2** |  |
| The authors have mostly addressed my comments, though there remain errors that I pointed out in my first review that have not yet been addressed:  1) The methods state on line 122 that individuals >1 were included, but on line 103 that individuals >4 were included. Which is correct?  2) Numerical inconsistencies remain. The sample sizes in Table 4 are not consistent with the sample sizes in Table 3. In addition, the sample sizes in Table 5 do not match the sample sizes in Table 1 and are substantially smaller. Are these errors in data entry, or were participants lost to follow up? This needs to be clearly addressed in the paper. If this represents loss to follow up, include a description of how characteristics of the participants with missing data compare to those that remain to address potential biases.  Additional copy-editing is required: - There are some French words in the abstract line 49. | In line 122, the sentence states that the study targeted individuals aged >1 during the 2017 OCV vaccination campaign, which in 2021 would be minimum 4 years old (thus indivuduals >4 during the current study period were included).  2) Inconsistency is due to lost on follow-up (as presented in the consort flow diagram-Figure 3). The number of participants dropping out from each follow up day is presented in figure 2.    The manuscript was revised to correct English mistakes throughout the text, thanks. |
| Reviewer 3 |  |
| The manuscript shows general improvement through revision; however, minor corrections are still required. Recommend to perform an overall check of the words used, the numbers mentioned, and table formatting. A few minor comments are provided in the attached PDF. | Thank you, the manuscript was improved and your comments addressed. |
|  |  |
| Figure Resubmissions:  While revising your submission, we strongly recommend that you use PLOS’s NAAS tool (<https://ngplosjournals.pagemajik.ai/artanalysis>) to test your figure files. NAAS can convert your figure files to the TIFF file type and meet basic requirements (such as print size, resolution), or provide you with a report on issues that do not meet our requirements and that NAAS cannot fix. | TIFF figures file types were resubmitted. |
